# A systematic review on hand gesture recognition techniques, challenges and applications

**DOI:** 10.7717/peerj-cs.218

**Published:** 2019-09-16

**Authors:** Mais Yasen, Shaidah Jusoh

**Affiliations:** Department of Computer Science, Princess Sumaya University for Technology, Amman, Jordan

**Keywords:** Hand gesture, Hand gesture applications, Hand gesture recognition challenges, Recognition techniques, Recognition

## Abstract

**Background:**

With the development of today’s technology, and as humans tend to naturally use hand gestures in their communication process to clarify their intentions, hand gesture recognition is considered to be an important part of Human Computer Interaction (HCI), which gives computers the ability of capturing and interpreting hand gestures, and executing commands afterwards. The aim of this study is to perform a systematic literature review for identifying the most prominent techniques, applications and challenges in hand gesture recognition.

**Methodology:**

To conduct this systematic review, we have screened 560 papers retrieved from IEEE Explore published from the year 2016 to 2018, in the searching process keywords such as “hand gesture recognition” and “hand gesture techniques” have been used. However, to focus the scope of the study 465 papers have been excluded. Only the most relevant hand gesture recognition works to the research questions, and the well-organized papers have been studied.

**Results:**

The results of this paper can be summarized as the following; the surface electromyography (sEMG) sensors with wearable hand gesture devices were the most acquisition tool used in the work studied, also Artificial Neural Network (ANN) was the most applied classifier, the most popular application was using hand gestures for sign language, the dominant environmental surrounding factor that affected the accuracy was the background color, and finally the problem of overfitting in the datasets was highly experienced.

**Conclusions:**

The paper will discuss the gesture acquisition methods, the feature extraction process, the classification of hand gestures, the applications that were recently proposed, the challenges that face researchers in the hand gesture recognition process, and the future of hand gesture recognition. We shall also introduce the most recent research from the year 2016 to the year 2018 in the field of hand gesture recognition for the first time.

## Introduction

**Figure 1:**
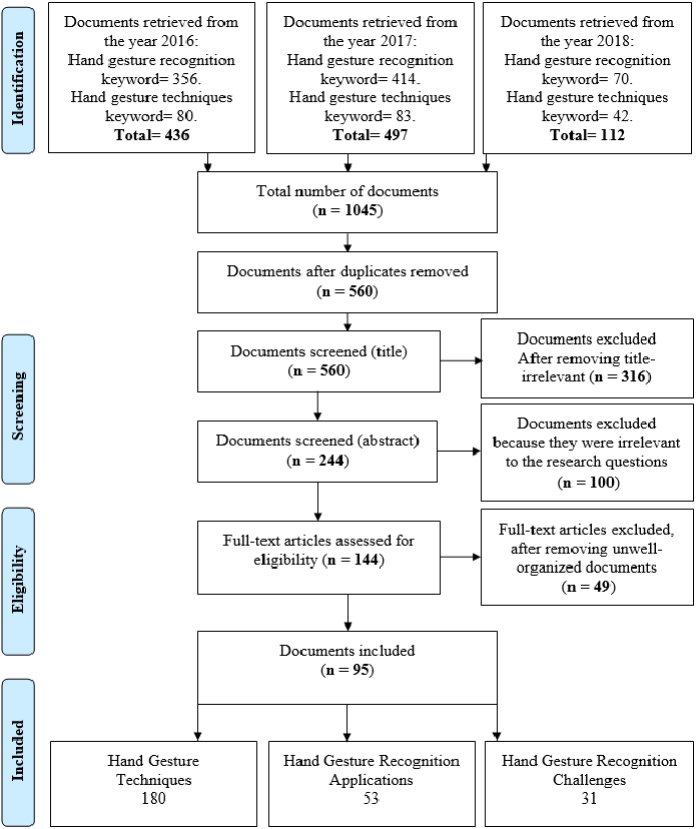
PRISMA flow diagram of the study selection process.

**Figure 2:**
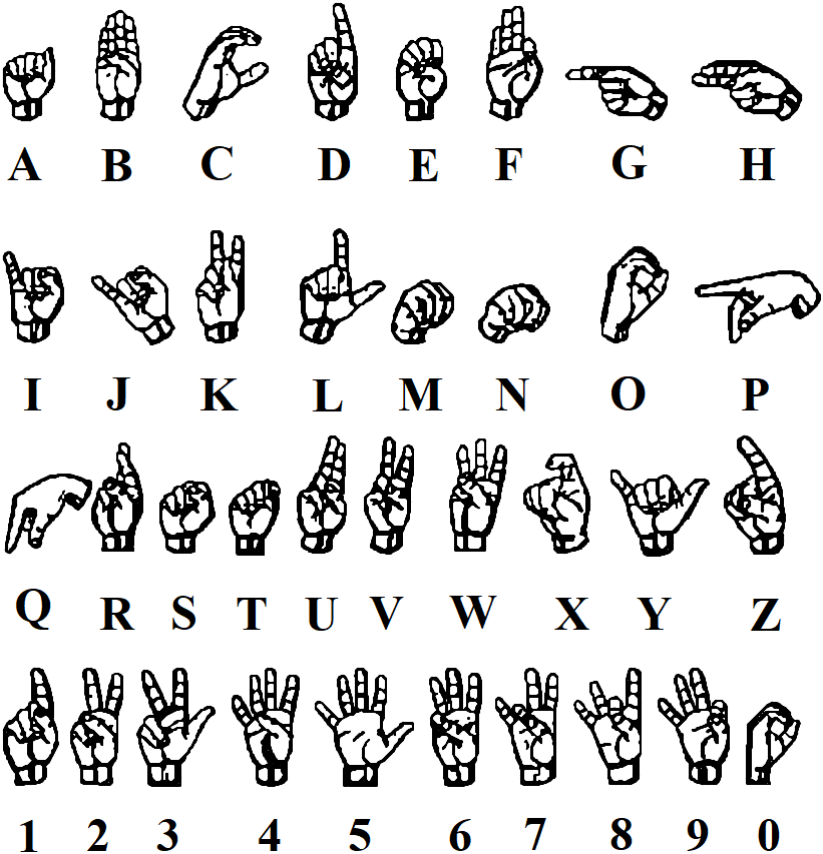
Examples of hand gestures in sign language (Rosalina et al., 2017).

A summary of the identification and selection of articles for inclusion in this review is presented in Fig. 1, according to the PRISMA statement (Moher et al., 2009). Hand gestures are a non-verbal method of communication using the movements of the human hand. This method is used either on its own or with another parallel communication method such as speech (Adam, 2004). The movements of a hand on the air as shown in Fig. 2 (Rosalina et al., 2017), is an example of a sign language using hand gestures. The representation of hand gestures is varying from reflecting a certain action to delivering a message that actually has a meaning (Adam, 2004). Hand Gestures are considered to be culture dependent, which means one gesture can range from giving a complimentary meaning to a giving a highly offensive meaning (Adam, 1994). Hand gestures are widely distributed on different kinds of applications, ranging from applications that are connected to the safety of humans, such as using hand gestures in controlling and directing flights operations (landing and taking off), to applications that are made for pleasure purposes, such as using it in gaming (Mohini et al., 2015).

With the development of today’s technology, and as humans tend to naturally use hand gestures in their communication process to clarify their intentions, hand gestures can play an important role in interchanging information between humans and computers (Aashni et al., 2017).

In Human–Computer Interaction (HCI), hand gestures have a wide number of applications that can guarantee the speed of communicating with the computer, give a user-friendly and aesthetic environment that would attract users, provide a nonphysical contact with the computer from a distance for user comfort and safety, and control complex and virtual environments in a much easier approach (Andrea, 2014).

On the other hand, hand gesture applications demand the user to be an expert and well trained at employing and understanding the meaning of different gestures (Samata & Kinage, 2015). There are many possible numbers of hand gestures; therefore, for each application, a different group of gestures is used to perform its operations. Also, from the computer perspective, the performance of recognizing hand gestures is affected by the environmental surroundings (such as light, background, distance, skin color) and the position and direction of the hand (Vaibhavi et al., 2014).

Perceptual computing is providing the computers with the ability of knowing what is happening around it (Bradley, 2015). In other words, the computer is capable of recognizing the different users and the different environmental factors occurring and existing in its surrounding. With that being said, hand gesture recognition is a type of perceptual computing user interface, that is used in HCI to give the computers the capability of capturing and interpreting hand gestures, and executing commands based on an understanding made on a certain gesture (Meenakshi & Pawan Singh, 2011).

Hand gesture recognition technique steps vary from simple to complex applications. Generally, the steps are usually divided as the following: first hand gesture frame acquisition, then hand tracking, then feature extraction, and finally classification to reach the output gesture.

Hand gesture frame acquisition is to capture the human hand gesture by the computer (Ananya, Anjan Kumar & Kandarpa Kumar, 2015). Whereas hand tracking is the ability of the computer to trace the hand and separate it from the background and from the surrounding objects (Ananya, Anjan Kumar & Kandarpa Kumar, 2015). The features needed to be extracted changes from one application to another, some of the features that could be taken into consideration are: fingers status, thumb status, skin color, alignments of fingers, and the palm position (Ananya, Anjan Kumar & Kandarpa Kumar, 2015). In artificial intelligence, machine learning enables the computers to learn without being explicitly programmed (Margaret, 2016). There are two types of learning; the process when algorithms reflect the gestures that has been learned in the past in training to new gestures is called supervised machine learning, and the process when algorithms draw inferences from the gestures is called unsupervised machine learning. Classification aims of building a model to classify new hand gestures based on previous training gestures.

The research work in hand gesture recognition has been developing for more than 38 years (Prashan, 2014). In 1977, a system that detects the number of fingers bending using a hand glove was proposed by Zimmerman (Praveen & Shreya, 2015). Furthermore, Gary Grimes in 1983 developed a system for determining whether the thumb is touching another part of the hand or fingers (Laura, Angelo & Paolo, 2008). In 1990, despite the limited computer processing powers, the systems developed then gave promising results (Prashan, 2014).

The field of hand gesture recognition is very wide, and a big amount of work was conducted in the last 2 to 3 years. In this research, we survey the latest researches that were done on hand gesture recognition. We shall also compare the different techniques, applications, and challenges presented by the surveyed work. The reason why the most recent research articles from IEEE database were chosen to be studied is that we wanted to construct a valid base of the current situation and technologies of hand gesture recognition. Furthermore, the articles published by IEEE in the year of 2016 to 2018 were considered to increase the intensity and focus of this study, and because the recent works were not sufficiently studied before, where the older ones were studied and compared more such as in Rafiqul & Noor (2012), Arpita, Sanyal & Majumder (2013) and Deepali & Chitode (2012). The contribution of this research can be summarized as the following:

1. Introducing the most recent researches from the year 2016 to the year 2018 in the field of hand gesture recognition for the first time.
2. Comparing the different techniques proposed, applications applied, and challenges discussed in the current available technology of hand gestures recognition.

The paper is structured as follows: Survey methodology section describes the research questions and methods used. The following section includes the results and analysis of the work studied. Then the next section discusses the future of hand gesture recognition. The last section concludes the research.

## Survey Methodology

This review is guided by the following three research questions:

1. What are the techniques used in hand gesture recognition?
2. What are the domains and applications that make use of hand gesture recognition?
3. What are the challenges in hand gesture recognition applications?

The first step of this systematic literature review is gathering all retrieved documents from the year 2016 to the year 2018, where the process of screening includes downloading the papers published on IEEE Explore, and reading their titles and abstracts. Two main keywords were used for the search process: hand gesture recognition and hand gesture techniques, other keywords were also used (such as hand gestures, hand gesture systems, hand gesture classification, hand gesture feature extraction, and sign language recognition) to include synonyms, where the keywords focused on the domain of research questions. The search process was performed by both authors (Mais Yasen and Shaidah Jusoh). A summary of the identification and selection of articles for inclusion in this review is presented in Fig. 1, according to the PRISMA statement (Moher et al., 2009). Literature included 1,045 studies. After removing duplicates, 560 titles were screened. The duplication occurs because the same work was retrieved more than once when multiple keywords were used. Then, 316 papers were excluded for having titles with no relevance to the review questions in general, where titles were scanned and processed by all authors to specify whether the works are pertinent to any chosen subcategory of hand gesture recognition. The next step was screening abstracts of all 244 retrieved documents. All 244 papers were reviewed and evaluated in reference to the research questions, where authors read the abstracts and evaluated whether they can find an answer to any of the core questions of this review, for example some of the papers excluded were discussing the electrical aspect of hand gesture acquisition methods, this process excluded 100 papers out of 244. Full papers of 144 potentially relevant references were selected for further examination. After reading the full papers, 49 poorly organized documents that do not have a clear methodology justification were removed due to lack of validation and weakness of results justification; for example, some of the works excluded did not explain why they chose their approach of acquisition or classification in comparison with all the possible approaches available in the recent technology, or did not explain why their proposed approach performed in the way they provided. Only 95 papers were selected to be included in this paper. Overall, the selection was made by the authors based on two criteria; relevance to the research questions, organization and form of writing of the papers studied. Classification of the selected paper is demonstrated in Table 1 which also shows the number of papers (intersected) included in each class and subclass. 180 papers were relevant to Hand Gesture Techniques, 53 papers were relevant to Hand Gesture Recognition Applications, and 31 papers were relevant to Hand Gesture Recognition Challenges.

**Table 1:** Classification of extracted papers.

Topic	Sub-topics	References count
Hand gesture techniques	Gesture acquisition methods	58
	Feature extraction	51
	Classification of hand gestures	71
Hand gesture recognition applications	Sign language	22
	Robotics	11
	Others	20
Hand gesture recognition challenges	Environmental surroundings	18
	Training and testing data	13

## Results and Analysis

**Figure 3:**
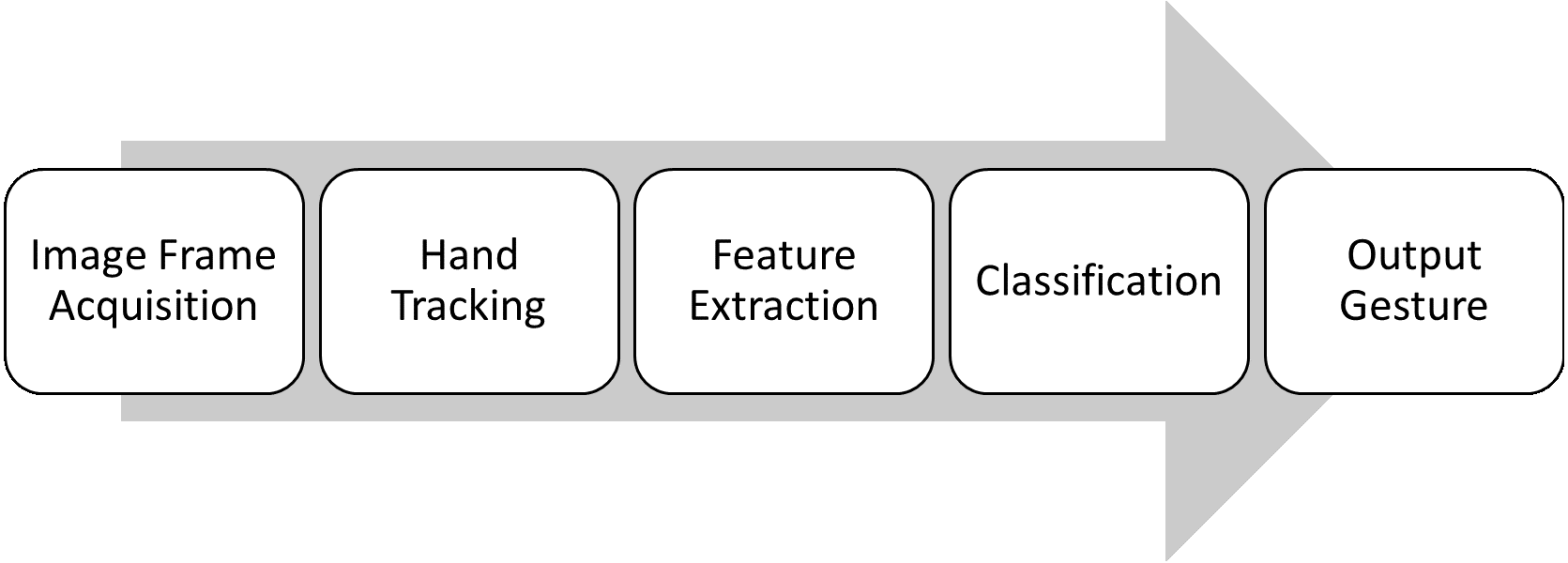
The basic steps of hand gesture recognition.

The procedure of hand gesture recognition is conducted by executing several steps as illustrated in Fig. 3; image frame acquisition or gesture acquisition is to capture the human hand gesture image by the computer (Ananya, Anjan Kumar & Kandarpa Kumar, 2015). This could be done using vision-based recognition where no special gadgets are required, and a web camera or a depth camera is used, furthermore special tools can be utilized such as wired or wireless gloves that detect the movements of the user hand, and motion sensing input devices (Kinect from Microsoft, Leap Motion, etc.) that captures the hand gestures and motions.

Hand tracking process is the ability of the computer to trace the user hand and separate it from the background and from the surrounding objects (Ananya, Anjan Kumar & Kandarpa Kumar, 2015). This can be done using multi-scale color feature hierarchies that gives the users hand and the background different shades of colors to be able to identify and remove the background, or by using clustering algorithms that are capable of treating each finger as a cluster and removing the empty spaces in-between them.

The features extracted change from one application to another, some of the features that could be taken into consideration are: fingers status, thumb status, skin color, alignments of fingers, and the palm position (Ananya, Anjan Kumar & Kandarpa Kumar, 2015). These features and other features can be extracted using several methods available, such as Fourier descriptor method which captures the palm, the fingers and the finger tips, or centroid method which captures the essential structure of the hand.

**Figure 4:**
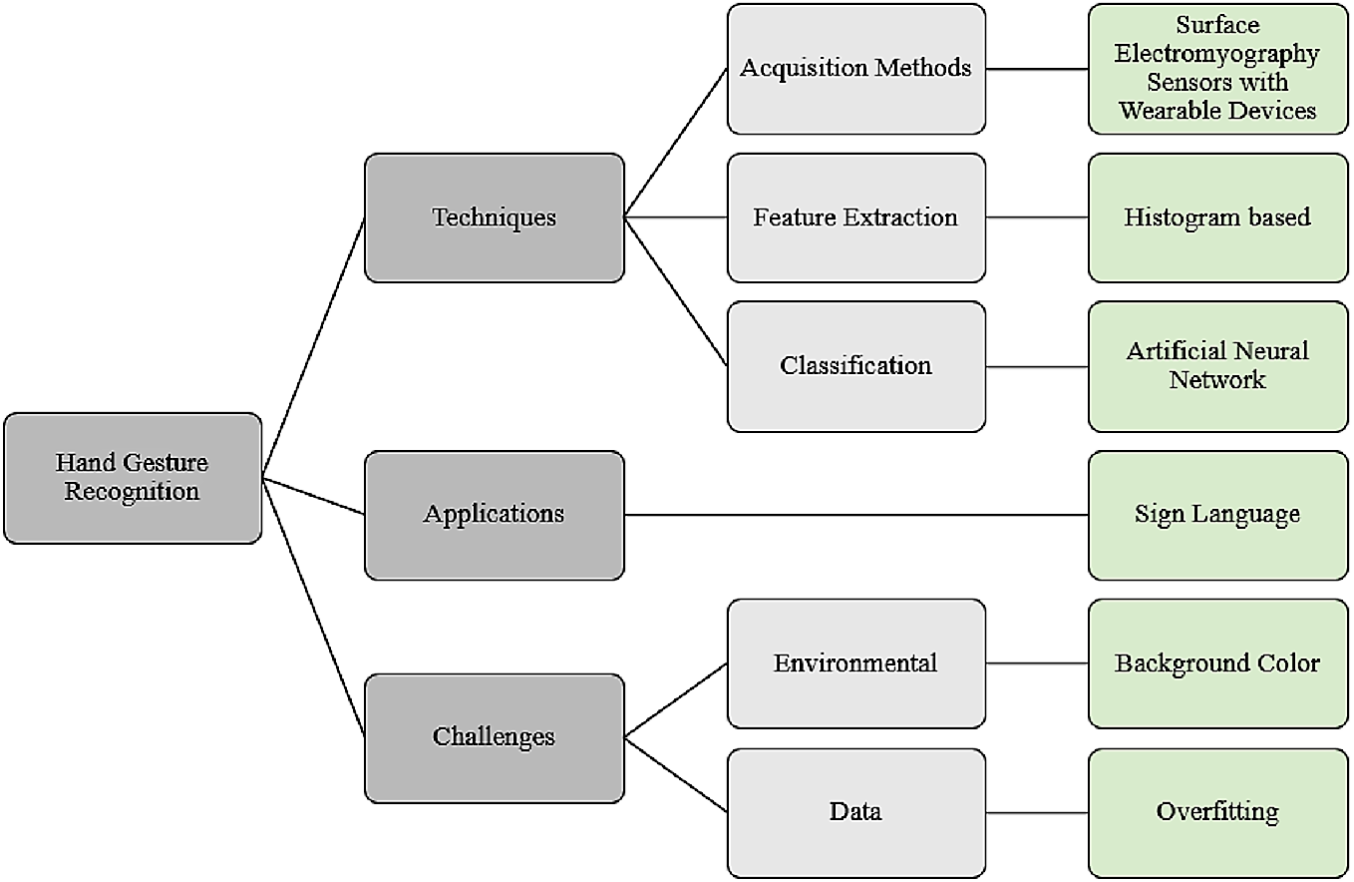
Results. Each research question is illustrated in this figure, along with the suggested subcategories and the most common technique or problem resulting in them.

The features extracted are then sent to training and testing the classification algorithm (such as Artificial Neural Networks (ANN), K-nearest neighbor (KNN), Naive Bayes (NB), Support Vector Machine (SVM), etc.) to reach the output gesture, for example the output gesture in a simple case can contain two classes to detect only two gestures such as open and closed hand gestures. In this section, we will be discussing the papers that were extracted in reference to the research questions in details, where Fig. 4 demonstrates the results of this review, answering each research question by showing the most popular method used in each subcategory.

### Hand gesture recognition techniques

The focus of our review in this section is on different gesture acquisition methods that has been used, the features considered for different applications and the feature extraction methods applied on the images to abstract those features, and finally the classification algorithms that has been recently used for classifying hand gestures.

#### Gesture acquisition methods

**Figure 5:**
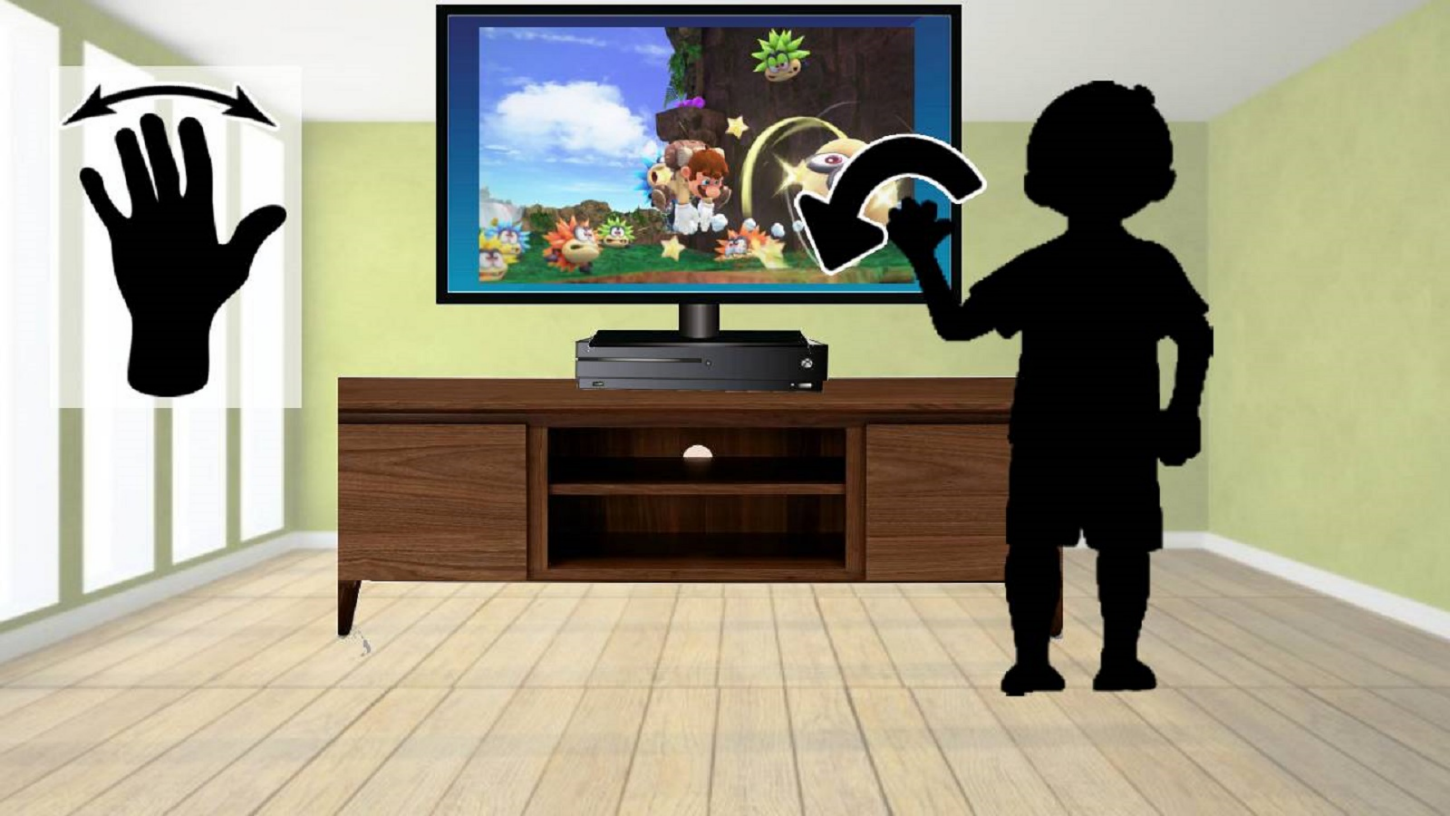
Example of hand gesture recognition using Kinect gestures (Microsoft, 2019).

Image hand gesture acquisition, as illustrated in Fig. 5 from Microsoft (2019) is to capture the human hand gesture image by the computer (Ananya, Anjan Kumar & Kandarpa Kumar, 2015). This could be done using vision-based recognition where no special gadgets are required and a web camera or a depth camera is used, furthermore special tools can be utilized such as wired or wireless gloves that detect the movements of the user hand, and motion sensing input devices (Kinect from Microsoft, Leap Motion, etc.) that capture the hand gestures and motions as showed in Fig. 5.

Several methods for hand gestures acquisition were discussed in the work studied using different acquisition tools, part of them used images that were captured in real time and others used previous images that were recorded in databases. Another classification to be considered is the nature of the acquisition which can be either a static hand gesture recognition where the gestures are represented by a constant image, or a dynamic hand gesture recognition were gestures are represented by the movement of the hand.

The first method is using vision-based hand gesture recognition to extract images which was proposed by Weiguo et al. (2017), Ananyaa et al. (2017), Alvi, Fatema & Mohammad (2016), Shome (2017). Where in Weiguo et al. (2017) it was built in a real-time system.

In Oinam et al. (2017) the authors implemented two different techniques of vision-based hand gesture recognition and one data glove-based technique. The vision-based techniques are static hand and real-time hand gesture recognition techniques. In data glove-based technique the glove had five flex sensors. Results showed that the vision-based technique was more stable and reliable compared to the data glove-based technique. Hand gestures are obtained by evaluating the contour captured from the image segmentation using a glove worn by the speaker in Rosalina et al. (2017). Also, in Danling, Yuanlong & Huaping (2016) they used a novel data glove called YoBu to collect data for gesture recognition.

The work in Gunawardane & Nimali (2017) compared using a data glove to track the motion of the human hand using flex sensors, gyroscopes and vision data with Leap Motion Controller. Results showed that the Leap Motion Controller had a high repeatability and high potential for soft finger type applications. Furthermore, in Eko, Surya & Rafiidha (2017), Shaun et al. (2017) and Deepali & Milind (2016) the gestures are also captured using Leap Motion Controller.

In Siji Rani, Dhrisya & Ahalyadas (2017) they used a new Hand Gesture Control in Augmented Reality System (HGCARS) where the gesture recognition is performed using a secondary camera and the reality is captured using an IP camera, the virtual object is added to the video feed obtained from an IP camera and controlled by using the position and depth of hand, measured using a webcam. Moreover, the authors of Salunke & Bharkad (2017), Rokhsana et al. (2017), Jessie et al. (2016) and Anshal, Heidy & Emmanuel (2017) used webcams for gathering input data.

In Aditya et al. (2017), a hand gesture recognition on top-view hand images observed by a Time of Flight (ToF) camera in a car for touchless interactions inside a car was proposed. The help of image capturing devices installed on computer was implemented in Nilima & Patil (2017). The authors of Hafız et al. (2017) presented a CMSWVHG (Control MS Windows via hand Gesture) to perform numerous windows actions using hand gestures using internal or external camera for taking input with the help of OpenCV. To control OS on the projected screen for a virtual mouse system without any hardware requirement one camera source was required (Rishabh et al., 2016). Also, data acquisition was done using Camera interfacing in Ashish & Aarti (2016a) and Ashish & Aarti (2016b).

To detect static hand gesture RGB cameras and depth data were used by Rytis & Arunas (2017) and Chenyang, Xin & Lianwen (2017), whereas in Chenyang, Xin & Lianwen (2017) they tested their approach on Sheffield Kinect Gesture (SKIG). A static hand gesture recognition using Kinect depth sensor to capture color, and infrared and depth frames was applied by Fuchang & Hijian (2016), Xiang, Lei & Qiang (2017), Jozef & Slavomír (2017), Sih-Huei et al. (2017) and Yiwen et al. (2017). Moreover, the authors proposed a Kinect based real time dynamic hand gesture recognition technique in Atharva & Apurv (2017), Devendrakumar & Kakade (2016) and Karthik et al. (2017).

A real time wearable hand gesture recognition system, which decoded the information from surface electromyography (sEMG) was proposed in Jian et al. (2017), Jinxing, Jianhong & Jun (2017), David & Donghan (2018), Kuang-Yow et al. (2017), Jakub, Tomasz & Piotr (2017), Shunzhan et al. (2017) and Andrés & Marco (2017). Also, a real time system based on the EMG with the surface electromyography measured by the commercial sensor Myo which is an armband placed on the forearm was proposed by Marco et al. (2017a), Marco et al. (2017b), Karthik et al. (2017) and Stefano et al. (2018).

In Yuhui, Shuo & Peter (2018), Yuta et al. (2017), Donq-Liang & Wei-Shiuan (2018), Ananta & Piyanuch (2016) and Nabeel, Rosa & Chan (2017) a sensor-based wearable wristband was presented for static hand gestures. The authors in Hari et al. (2016) and Erhan, Hakan & Baran (2017) presented making use of the sensors in smartphones. The authors of Yifan et al. (2018) used wearable devices such as VR/AR helmet and glasses in a gesture recognition system. A Doppler radar sensor with dual receiving channels was used to acquire a big database of hand gestures signals in Jiajun & Zhiguo (2017). A gesture recognition system for human–computer interaction based on 24 GHz radars in Shengchang et al. (2017) and 2.4-GHz continuous radar in Takuya et al. (2017) were used.

Moreover, an interface based on a mechanomyography (MMG) signal to capture arm movement and hand gesture was applied in Yuntao et al. (2017) and Piotr, Tomasz & Jakub (2017).

The work of Ibrahim et al. (2018) proposed a hand gesture system using two monopole antennas to measure signatures at different locations, and the work of Zengshan et al. (2018) developed a wireless sensing WiCatch, where the motion locus of the gesture is developed by constructing virtual antenna array based on signal samples.

#### Feature extraction

Hand tracking process is the ability of the computer to trace the user hand and separate it from the background and from the surrounding objects (Ananya, Anjan Kumar & Kandarpa Kumar, 2015). This can be done using multi-scale color feature hierarchies that gives the users hand and the background different shades of colors to be able to identify and remove the background, or by using clustering algorithms that are capable of treating each finger as a cluster and removing the empty spaces in-between them. In this subsection, we will be discussing the different features used by the different applications implemented in the reviewed work. Furthermore, the feature extraction methods used will also be discussed.

##### Feature extraction methods.

Feature extraction methods are used in order to extract the useful information from the images that helps in the recognition process of gestures. These features can be extracted using several methods available, such as Fourier descriptor method which captures the palm, the fingers and the finger tips, or centroid method which captures the essential structure of the hand.

The work of Haitham, Alaa & Sabah (2017) applied two methods of feature extraction, a contour hand and complex Alhzat. In Weiguo et al. (2017) feature extraction module was applied using Discrete Fourier Transform (DFT) operations of histograms (vertical and horizontal). The proposed approach of Salunke & Bharkad (2017) took the input data from a portable webcam then processed the images and after that extracted a histogram of gradients features. The objective of Himadri et al. (2018) was to do segmentation of the hands using polygon approximation and approximate convex decomposition, then record the unique features between various convex segments of the hand as a method of feature extraction.

Regional Contrast (RC) based salient object extraction algorithm, and a method using the color statistics of image were used in Qingrui et al. (2017). To detect the start and end points of gestures a gesture spotting algorithm was applied in Hari et al. (2016). Experiments of Chenyang, Yingli & Matt (2016) followed five different feature extraction strategies; depth image sequence, body joints & facial landmarks, hand shapes & facial expressions/attributes. In Jose et al. (2017), digital image processing techniques were used to eliminate noise, to improve the contrast under different illumination, to separate the hand from the background and to cut the region containing the hand.

Histogram of Oriented Gradients (HOG) was used in Vimonhak, Aranya & Somsak (2017) for image processing to extract characteristics of the hand. In the work of NilimaS2017, the Accurate End Point Identification method was implemented and applied on gesture images which were captured in varying backgrounds to detect edge points and branch points and it was also applied on blurred images containing multiple objects. The authors of Isack, Zhongfu & Jamal (2017) employed higher order local autocorrelation (HLAC) feature extraction method.

The authors of Xi, Chen & Lihua (2017) proposed using Wavelet Invariant Moments, the hand region was separated based on the depth information, and the wavelet feature was calculated by enforcing the wavelet invariant moments of the hand region, and the distance feature was extracted by calculating the distance from fingers to hand centroid.

Feature extraction like time, frequency and time-frequency were used in Jiajun & Zhiguo (2017) and Andrés & Marco (2017). Authors of Chenyang, Xin & Lianwen (2017) used a robust feature, path signature (PS) and its compressed version, log path signature (LPS) to extract effective features of hand gestures. Discrete wavelet transformation and singular value decomposition were used for features extraction, a genetic algorithm with effective fitness function was used to select optimal features by eliminating redundant and irrelevant features for improving the recognition performance of the system in Rasel et al. (2017).

In Rania, Mohammed & Mahmoud (2017) system for HGR that is based on dimensionality reducing the histogram of oriented gradients feature vectors by applying principal component analysis was implemented. A hand gesture recognition method based on salient feature point selection was proposed in Yiwen et al. (2017), the shape feature of hand gesture was extracted from the contour, and the salient feature points were selected by a new algorithm to represent the hand gesture.

In experiments of Vivek, Ramesh & Kagalkar (2017) image processing techniques like outline investigating based on edge recognition, wavelet change, disintegration, widening, obscure disposal, and commotion end were used, they also used Histogram Orientation Gradient called HOG for shape highlight extraction and most vital part investigation for list of capabilities streamlining and diminishment.

They also used a polygonal shape approximation strategy with a special chain-coding for shape-similarity matching in Oyndrila et al. (2016). In Vladislava, Predrag & Goran (2016) recognition was for 10 hand gestures, images were captured on two different backgrounds and with several space orientations, and histogram of Oriented Gradients method was applied for feature extraction. The Gaussian Mixture Models (GMM) based on human skin pixels and tracks segmented foreground using optical flow to detect hand swipe direction was proposed in Srinidhi et al. (2016).

Authors of Ananta & Piyanuch (2016) employed Daubechies wavelet transforms for feature extraction. In Rajeshree & Jayashree (2016) they proposed a new method to find flow of hand for special signs using chain code, and recognition technique of signs (Dynamic digits) that is independent of size and color of hand using binary images. In Ashish & Aarti (2016a) and Ashish & Aarti (2016b) feature extraction was done using Blob Detection and Contour extraction. In Jessie et al. (2016) image processing techniques such as: color segmentation, visual-hand tracking, pre-processing, and feature extraction were used.

In Ashish & Aarti (2016a) and Ashish & Aarti (2016b) features were extracted using Color jiltering and skin segmentation, convexity hull algorithm was implemented just for finger point detection and number recognition. Where in Lalit & Pritee (2017), an acquisition module that spots the legitimate gesture trajectory by implementing pen-up and pen-down actions using depth thresholding and velocity tracking was proposed, the extracted trajectory is recognized using the equipolar signature (EPS) technique.

Conventional method was used in Devendrakumar & Kakade (2016) to utilize separation of hand from surrounding environment and then find palm points. Each hand part in Shome (2017) was modeled as a convex hull and pairwise distance between the parts was calculated using GJK-EPA algorithm. Principal Component Analysis (PCA) was used in Marlon, Alistair & Mohamed (2016) to reduce the dimensionality and extract features of images of the human hand.

The work in Anshal, Heidy & Emmanuel (2017) processed the hand gesture image by combining image segmentation and edge detection to extract morphological information, and frames were processed using the multi-modality technique used for processing individual characters. In Vedha Viyas et al. (2017) to improve recognition rate of hand gestures, various image processing techniques were used such as Histogram Equalization, Median filtering, Average filtering, and Morphological filtering, feature extraction image matching was done using cross-correlation co-efficient.

The proposed techniques in Riya et al. (2017) relied on multiple representations namely HOG, GIST and BSIF, they used feature fusion which is the process of combining two feature vectors to obtain a single feature vector. Extraction of a series of features based on convex defect detection a model was proposed in Soukaina et al. (2018), taking advantage of the close relationship of convex defect and fingertips.

##### Features extracted.

The features extracted change from one application to another, some of the features that could be taken into consideration are: fingers status, thumb status, skin color, alignments of fingers, and the palm position (Ananya, Anjan Kumar & Kandarpa Kumar, 2015).

There are several features that could be considered and extracted from the hand gestures which are highly dependent on the application. Skin color feature was used in Weiguo et al. (2017), Xiang, Lei & Qiang (2017), Rokhsana et al. (2017). Hand gestures in Himadri et al. (2018) were represented by hand shapes, orientations and movement of the hands, alignments of the fingers, and the palm position. In David & Donghan (2018) open hand, wrist radial deviation, ulnar deviation, wrist extension, wrist flexion, and closed fist were considered. Scale, rotation, translation, illumination, noise and background were extracted in the work of Jose et al. (2017). In Yuta et al. (2017) finger movements were observed because the muscles and bones on the back of the hand are linked to the fingers, also skin deformation was measured by the distance between the device and skin with sensors.

The classifier in Piotr, Tomasz & Jakub (2017) had five gestures (fist, pronation, supination, flexion, extension) and the feature vector consisted of 18 features: five representing muscle activity (RMS) and 13 parameters corresponding to relative sensor orientation. A feature vector which was composed of wavelet invariant moments and distance feature was generated by the work of Xi, Chen & Lihua (2017). The authors of Shunzhan et al. (2017) extract five eigenvalues in the time domain. Geometric features such as area and centroid were extracted from each video frame to capture the trajectory of the moving hand and compare it with the training gestures in the experiments of Atharva & Apurv (2017).

The features used in Gunawardane & Nimali (2017) were: position, orientation, velocity and acceleration, bending angle of the fingers. To represent the global movement of hand skeleton the global motion features were extracted by Xinghao et al. (2017). In experiments of Sih-Huei et al. (2017) the hand region is recognized by using information about the skeleton of the segmented depth images, then a histogram of the oriented normal 4D (HON4D) and a histogram of oriented gradient (HOG) were extracted to represent the motion patterns.

For each hand gesture type, occlusion, light, shadow and background were considered as features in Biyao et al. (2017), whereas the work of Anna, Sung & Jae-Gon (2018) presented a detection method that utilizes depth image obtained by incoming stereo image sequences and skin color information in a combined way, then a detected hand contours based on Bezier curve to provide an interoperable interface between a detection module and a recognition module was also presented, and a set of hand gestures with a combination of open fingers and rotational angles were used for the hand gesture recognition of their system.

The proposed system (Oyndrila et al., 2016) identified hand-palm in video stream based on skin color and background subtraction scheme. Where in Rishabh et al. (2016) glove tracking was done and then fingertips were detected with respect to centroid of the hand. Features used in Ashish & Aarti (2016a) and Ashish & Aarti (2016b) were orientation, Centre of mass centroid, fingers status, thumb in positions of raised or folded fingers of hand. On the other hand, features extracted in Soumya & Muzameel (2017) were size, shape, color or texture.

Flexion, extension, abduction and grasp of forearm muscles features were used to detect four different movements of the wrist (Stefano et al., 2018). The features had to be able to characterize gestures, and invariant under translation and rotation of hand gesture to ensure reliable recognition in Soukaina et al. (2018).

#### Classification of hand gestures

The features extracted are sent to training and testing the classification algorithm (such as Artificial Neural Networks (ANN), K-nearest neighbor (KNN), Naive Bayes (NB), Support Vector Machine (SVM), etc.) to reach the output gesture, for example the output gesture in a simple case can contain two classes to detect only two gestures such as open and closed hand gestures, or in sign language as shown in Fig. 2.

The classification of the information extracted from the images of hand gestures is required to be able to recognize these gestures by the computer, there are several classification algorithms that can be used for this purpose.

The work of Haitham, Alaa & Sabah (2017), Weiguo et al. (2017), Rosalina et al. (2017), Jakub, Tomasz & Piotr (2017), Piotr, Tomasz & Jakub (2017), Shunzhan et al. (2017), Erhan, Hakan & Baran (2017), Vladislava, Predrag & Goran (2016), Deepali & Milind (2016) and Ananta & Piyanuch (2016) proposed using Artificial Neural Networks (ANN) for classification, whereas in Jakub, Tomasz & Piotr (2017) and Piotr, Tomasz & Jakub (2017) SoftMax output layer was used with feedforward ANN; in Shunzhan et al. (2017), Erhan, Hakan & Baran (2017), Alvi, Fatema & Mohammad (2016), Vladislava, Predrag & Goran (2016); Deepali & Milind (2016) and Ananta & Piyanuch (2016) backpropagation training methods were used, and the authors in Jessie et al. (2016) used Kohonen self-organizing maps as a type of ANN to classify data sets in unsupervised manner to convert hand gestures into Filipino words.

In Chun-Jen et al. (2017), a synthetically-trained neural network was used for 3D hand gesture identification. The training process of a deep-learning neural network required a large amount of training data. The authors of Chenyang, Xin & Lianwen (2017) proposed using the LPSNet, an end-to-end deep neural network for hand gesture recognition with novel log path signature features. In Yifan et al. (2018) and Biyao et al. (2017) they proposed using deep neural networks for classification.

The authors in Sungho & Wonyong (2016) came up with two dynamic hand gesture recognition techniques using low complexity recurrent neural network (RNN) algorithms for wearable devices, the first was based on video signal and uses convolutional neural network (CNN) with RNN for classification, and the other used accelerometer data and applied RNN for classification. In contrast, Xinghao et al. (2017) used a bidirectional recurrent neural network (RNN) with the skeleton sequence to augment the motion features for RNN were used.

Deep convolutional neural network was used for classification in Ibrahim et al. (2018), David & Donghan (2018), Javier, Paula & Robinson (2017), Jose et al. (2017), Aditya et al. (2017), Yuntao et al. (2017), Peijun et al. (2017), Jozef & Slavomír (2017), Jiajun & Zhiguo (2017), Takuya et al. (2017) and Fabian et al. (2017), where in Aditya et al. (2017) the authors aimed to improve the detection of hand-gestures by correcting the probability estimate of a Long-Short-Term Memory (LSTM) network by pose prediction performed by a Convolutional Neural Network (CNN). They used Principal Component Analysis (PCA) as a training procedure to reduce the labelled data of hand pose classification to perfect the initialization of weights for the CNN.

The authors in Fuchang & Hijian (2016), Salunke & Bharkad (2017) and Marlon, Alistair & Mohamed (2016) used K-nearest neighbor (KNN) classifier to recognize hand gestures. The work presented in Kuang-Yow et al. (2017) classified the recognition using k-Nearest Neighbor (KNN) and Decision Tree algorithms combination.

Support vector machine (SVM) was used for classification in Yuhui, Shuo & Peter (2018), Chenyang, Yingli & Matt (2016), Yuta et al. (2017) Xi, Chen & Lihua (2017), Rasel et al. (2017), Reshna & Jayaraju (2017), Zengshan et al. (2018), Ashish & Aarti (2016a), Ashish & Aarti (2016b), Zhiwen et al. (2017) and Stefano et al. (2018). Decision tree was used to classify the gestures in Shengchang et al. (2017). To recognize the patterns in Jian et al. (2017), they used a modified deep forest algorithm. In Shaun et al. (2017) and Riya et al. (2017), they used a random regression forest algorithm for classification.

In Jinxing, Jianhong & Jun (2017) the hand gesture was modeled and decomposed by the use of Gaussian Mixture Model-Hidden Markov Models (GMMHMM), GMMs are used as sub-states of HMMs to decode sEMG feature of gesture. Moreover, a hand gestures recognition system was implemented using the incorporation of Bhattacharyya divergence into Bayesian sensing hidden Markov models (BS-HMM) in Sih-Huei et al. (2017). Also, in Devendrakumar & Kakade (2016) Hidden Markov Model was used for classification.

The work of Hari et al. (2016), Atharva & Apurv (2017), Yiwen et al. (2017) and Washef, Kunal & Soma (2016) presented using the dynamic time warping algorithm for classification. Whereas Marco et al. (2017a) and Marco et al. (2017b) used the k-nearest neighbor rule together with the dynamic time warping algorithm for classification.

Naive Bayes was applied as the training method for classification of Eko, Surya & Rafiidha (2017). Multiple linear discriminant analysis (LDA) classifier was adopted to classify different hand gestures in Isack, Zhongfu & Jamal (2017) and Yuefeng et al. (2016).

Classification of digits gesture from 11 to 20 was done using Principal component analysis by Rajeshree & Jayashree (2016). The work (Danling, Yuanlong & Huaping, 2016) used extreme learning machine (ELM) for gesture recognition.

The work presented in Himadri et al. (2018) used Support Vector Machine (SVM), Artificial Neural Network, Naive Bayes and K-Nearest Neighbor (K-NN) classifiers as the training methods to recognize the features extracted. In contrast, in Ananyaa et al. (2017) the best classification accuracy was achieved using Euclidean distance and Eigen vector, but this result is for a very small dataset, and the best result was a dataset containing nearly 720 images that used Support vector machine for classification of images; also, using Artificial Neural Network provided an accuracy of 89.48%. Furthermore, Multi-class Support Vector Machine (SVM) and k-Nearest Neighbors (KNN) classifiers were used to classify the hand gestures in Rania, Mohammed & Mahmoud (2017), experiments showed that the accuracy of KNN classifier were better than SVM classifier. In Nabeel, Rosa & Chan (2017) Support Vector Machine (SVM), Decision Tree (DT), K-Nearest Neighbors (KNN), and Linear Discriminant Analysis (LDA) were compared in classification performance, and results showed that LDA got the highest accuracy.

### Hand gesture recognition applications

Hand gestures can be used as a natural communication with the computer; with today’s technologies, the number of applications that could apply hand gesture recognition is rapidly increasing. In this section, we will be discussing the most recent applications that were presented in the years 2016 to 2018.

#### Sign language

Sign languages, as illustrated in Fig. 2, use manual communication to explain a certain meaning. This can include using hand gestures, movements, fingers orientation to deliver a certain word or meaning.

The work of Weiguo et al. (2017), Chenyang, Yingli & Matt (2016), Rasel et al. (2017), Shaun et al. (2017), Deepali & Milind (2016), Nabeel, Rosa & Chan (2017); and Anshal, Heidy & Emmanuel (2017) proposed using hand gesture recognition for American Sign Language (ASL) for people who are hearing impaired, whereas in Shaun et al. (2017) they did not evaluate the letters that are dynamic (like: j and z). In Nabeel, Rosa & Chan (2017) 36 gestures were studied including 26 ASL alphabets and 10 ASL numbers. In Anshal, Heidy & Emmanuel (2017) two different translation paradigms; English characters (alphabet) and complete words or phrases were proposed.

An alternative to talking for deaf & dumb people who have hearing or speech problems using sign language recognition and hand gestures was proposed in Himadri et al. (2018), Oinam et al. (2017), Reshna & Jayaraju (2017), Vivek, Ramesh & Kagalkar (2017), Oyndrila et al. (2016), Ashish & Aarti (2016a), Ashish & Aarti (2016b) and Rishabh et al. (2016). Whereas in Rosalina et al. (2017) a sign language that consists of the alphabetic from “A” to “Z”, the numbers from “0” to “9”, and some additional punctuation marks like “Period”, “Question Mark”, and “Space” using static hand gesture recognition was produced.

The work of Jose et al. (2017) used the alphabet of sign language of Peru (LSP). Also, Vimonhak, Aranya & Somsak (2017) proposed a technique for the recognition of Lao alphabet sign language for the deaf people. In Eko, Surya & Rafiidha (2017), Indonesian Sign Language System (SIBI) was applied for normal people to learn sign language and communicate to people with hearing disability. Moreover, in Donq-Liang & Wei-Shiuan (2018), the authors used 29 Turkish fingerspelling signs. A framework for recognizing Sinhala sign language gestures and translating them in to natural language for hearing & speaking impaired people was proposed in Vladislava, Predrag & Goran (2016). The authors of Jessie et al. (2016) converted hand gestures into Filipino words. The work presented in Marlon, Alistair & Mohamed (2016) used the alphabet of Irish Sign Language. Finally, Indian Sign Language (ISL) hand gesture recognition system was introduced in Riya et al. (2017).

#### Robotics

**Figure 6:**
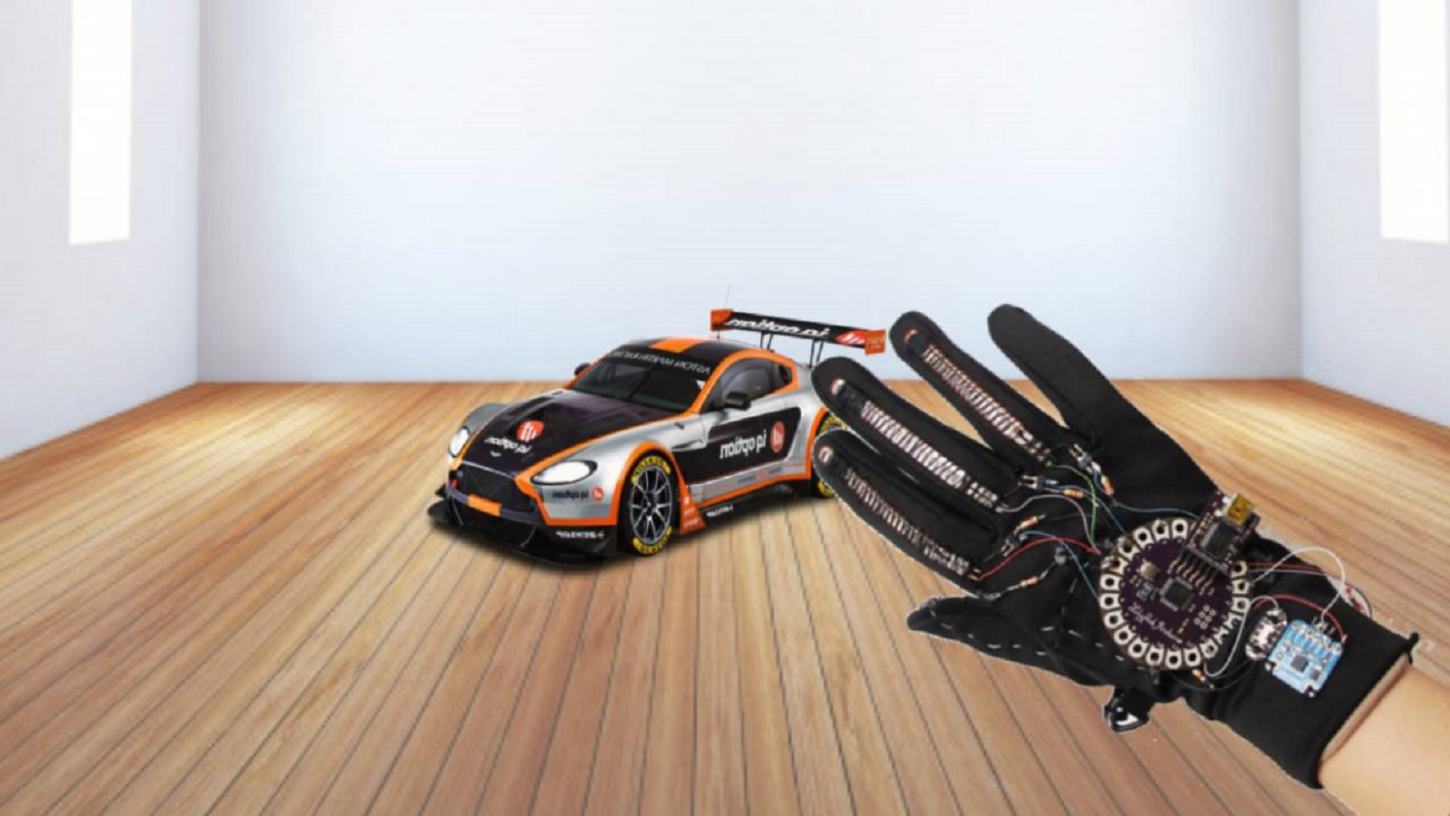
Example of the use of hand gestures in robotics (Elecbits, 2019).

Hand gestures can also be employed in the process of building robotics, as illustrated in Fig. 6 (Elecbits, 2019) which shows the use of a glove to control a robotic vehicle. A recognition frame of continuous hand gestures of upper-limb exoskeleton robot was proposed in Jinxing, Jianhong & Jun (2017). In Yuhui, Shuo & Peter (2018) they had three groups of hand gestures: six wrist gestures, five single finger flexions, and ten Chinese number gestures that were built to control a robotic arm. The authors of Sungho & Wonyong (2016) came up with two dynamic hand gesture recognition techniques using low complexity recurrent neural network (RNN) algorithms for a wearable device.

The experiments of Xiang, Lei & Qiang (2017) produced a control system for robotic wheelchair for the aged and the disabled, which contained two parts: gesture interaction and intelligent wheelchair. Also, the approach of Javier, Paula & Robinson (2017) allowed the consequent execution of a robotic agent for the delivery of objects. In Yuntao et al. (2017) the authors introduced a wearable sensor suite fusing arm motion and hand gesture recognition for operator control of UAVs.

The authors of Yuta et al. (2017) proposed a wearable device with photo-reflective sensors arranged in an array to recognize hand gestures by measuring the skin deformation of the back of the hand. Also, in Danling, Yuanlong & Huaping (2016) hand gestures recognition was used in human–robot interaction (HRI). In experiments of Devendrakumar & Kakade (2016), continuous trajectory path made by hand over a period of time was considered for real time dynamic gesture recognition purpose using Kinect sensor for controlling robotic arm. A hand gesture-based control design was proposed for mobile robots in Hang et al. (2017), where mobile robots can move according to the signals encoded by hand gestures. Work of Vedha Viyas et al. (2017) dealt with a robotic arm using hand gestures for many applications such as automation, medical applications and gaming.

#### Others

A static hand gesture recognition in a real-time human–computer interaction application was proposed in Salunke & Bharkad (2017) to control the Power Point presentations from a distance. A human–computer interaction based on hand gesture in a projector-camera system was presented in Qingrui et al. (2017). Also, real-time hand gesture recognition for flight control of multiple quadrotors was applied in David & Donghan (2018). The authors of Javier, Paula & Robinson (2017) presented the design of a convolutional neural network architecture using the MatConvNet library in order to achieve the recognition of 2 classes: “open” and “closed” and “unknown”. The approach studied in Marco et al. (2017a) and Marco et al. (2017b) could be used in multiple applications in medical and engineering fields.

The study of Aditya et al. (2017) showed hand gesture recognition on top-view hand images observed by a Time of Flight (ToF) camera in a car for touchless interactions inside a car. In Peijun et al. (2017) they used seven kinds of hand gestures that can command a consumer electronics device, such as mobile phones and TVs. The authors in Siji Rani, Dhrisya & Ahalyadas (2017) proposed an Augmented Reality (AR) to merge the virtual world into the real world and to enhance the perception of the real world by adding 2-D virtual objects.

The experiments of Yu et al. (2017) described a method of hand gesture recognition using Principle Component Analysis (PCA) implemented in Android phone. A real-time hand gesture recognition system was proposed using electromyography (EMG) in the field of medicine and engineering, with a higher number of gestures to recognize in Marco et al. (2017a) and Marco et al. (2017b). The work of Rokhsana et al. (2017) presented a hand gesture-based computer mouse control system. The authors of Hafız et al. (2017) presented a CMSWVHG (Control MS Windows via hand Gesture) to perform numerous windows actions using hand gestures.

The work discussed in Anna, Sung & Jae-Gon (2018) presented a method to detect and to recognize hand gestures for generating hand gesture-based commands to control the media consumption in smart glasses. Hand gesture recognition or finger angle prediction for Ultrasound imaging was proposed in Yuefeng et al. (2016). A hand gesture recognition technique on a smartphone using Google Cardboard (GC) and Wearality2 in phones was applied in Srinidhi et al. (2016).

A real-time hand gesture recognition technique for presentation was proposed in Rishabh et al. (2016), to control OS on the projected screen for a virtual mouse system without any hardware requirement. Hand-gesture-based commands were proposed in Lalit & Pritee (2017) to replace touch and electromechanical input panels using vision-based mid-air character input.

Gesture recognition system was proposed in Soumya & Muzameel (2017), Mudra is an expressive form of gesture that is mainly used in Indian classical dance form where the gesture is in visual form to connect with the audience. The authors in Zhiwen et al. (2017) presented a real-time hand gesture recognition by using Kinect sensor, to control mouse by user hands for operations such as ‘clicking’, ‘dragging’ and ‘dropping’, and engaged/disengaged gestures. Gesture recognition in Karthik et al. (2017) was used for Bio Robotics, the paper focused on presenting a sensor based human gesture recognition for the Hand Cricket game.

### Hand gesture recognition challenges

The process of hand gestures recognition consists of a set of complex steps that has many possible difficulties that could stand in the way of having accurate recognition. In this section, we will discuss the major environmental surroundings challenges and the training and testing dataset challenges that could occur.

#### Environmental surroundings

In Fuchang & Hijian (2016) the environmental background, light and rotation, translation and scale change proposed a difficulty; the authors used depth data to help in hand separation and to accomplish a synchronized color and depth images in Kinect. Different light brightness (dull, medium, and bright) conditions were tested in Salunke & Bharkad (2017) to overcome their problem, the results of the bright light level gave the best accuracy as expected. Furthermore, the results of Oinam et al. (2017) showed that the vision-based techniques gave an accuracy of 100% in bright lighting condition with a white background.

The work of Qingrui et al. (2017) showed that it was difficult to achieve proper accuracy when having complex backgrounds, variable external illumination, and shadows of hand gesture. The authors of Hari et al. (2016) presented a continuous hand gestures recognition technique using three-axis accelerometer and gyroscope sensors in smartphones, a gesture coding algorithm was also used to reduce the influence of unstableness of the user hand.

Digital image processing techniques were used in Jose et al. (2017) to eliminate noise, to improve the contrast under different illumination, to separate the hand from the background and to cut the region containing the hand, where results of images with complex background got low accuracy.

In Rytis & Arunas (2017) the authors suggested that RGB cameras can be used to detect hand, but it has limited applications because hand detection was hard in some lighting conditions or in different skin colors, using depth camera data was better in distinguishing hands in front of camera.

Study of Peijun et al. (2017) suggested that spatial localization of the hands when it contains background, human face, and human body could be a challenging task, where the results achieved an accuracy of 97.1% in the dataset with simple backgrounds and 85.3% in the dataset with complex backgrounds.

The authors of Yu et al. (2017) were capable of solving the problems that faced them such as different size of gesture image captured, different angle of gesture’s rotation and flipped gesture. After experiments of Atharva & Apurv (2017) they found that if there was a larger object than the hand that the Kinect captures, wrong results will be achieved. The results of Hafız et al. (2017) were highly affected by the background noise where they achieved an accuracy of 82.52%.

In experiments of Yifan et al. (2018) and to strengthen the recognition accuracy, and as the dataset used contained more than 24,000 gesture and 3,000,000 frames for both color and depth modalities from 50 subjects, they tested 83 different static and dynamic gestures with six diverse indoor and outdoor scenes respectively with variation in background and illumination, they also tested when people perform gestures while they are walking and they achieved an acceptable accuracy.

In Reshna & Jayaraju (2017) The authors used complex backgrounds with respect to the skin color, and results showed a disadvantage in this system which was the lighting conditions. The results of Rania, Mohammed & Mahmoud (2017) showed that the proposed algorithm achieved recognition rate of 97.69% under different hand poses and complex background with changes in lightning, moreover their system was robust to rotation, scale, translation, and lighting.

Also, the results of Yiwen et al. (2017) showed that their method was adjusting to translation, rotation scaling and articulated deformation. Time of Flight (ToF) data was used in Fabian et al. (2017) which tends to be overly noisy depending on various factors such as illumination, reflection coefficient and distance that were studied in their research process. The system of Washef, Kunal & Soma (2016) overcame the limitations of a glove-based approach and the vision-based approach concerning different illumination conditions, background complexity and distance from camera. The system in Lalit & Pritee (2017) was applicable for rotation, scale, and translation variations, directions, and the dataset contained digits, alphabets, and symbols.

#### Training and testing Data

The training and testing data problems can be classified as either having a small set of gestures that are insufficient for recognition training or having a large set of gestures that are imbalanced leading to a low recognition accuracy. The training data of Haitham, Alaa & Sabah (2017) had only 25 images and the results reached an accuracy of 82.2%. In Weiguo et al. (2017) they trained their system using 24 static American Sign Language (ASL) and their accuracy reached 93.3%, whereas in Fuchang & Hijian (2016) they used detailed features to avoid the problem of having imbalanced data and images.

To overcome the problem of having more than one meaning and move to represent a gesture the authors of Oinam et al. (2017) used vision-based hand gesture recognition systems and data glove-based hand gesture recognition systems. In Jian et al. (2017) 16 hand gestures include 10 dynamic and six static gestures were used, and results showed that gestures were correctly recognized and the accuracy was 96%.

The work in Ibrahim et al. (2018) tested the system using ten hand gestures achieving their goal which is to maximize the recognition accuracy using Pre-trained networks. The data of Javier, Paula & Robinson (2017) contained six architectures with variance in the hyperparameters and depth, their results achieved good accuracy. Classification of 24 hand gestures to improve the rate of recognition accuracy was proposed in Jose et al. (2017) reaching 96.20% for simple backgrounds.

In Aditya et al. (2017) five hand poses with nine class hand gestures were used reaching an accuracy of 89.50% as a result. The work of Vimonhak, Aranya & Somsak (2017) used 54 Lao alphabets in the experiments, testing data had 540 gestures from four individuals, it was difficult to maintain constant speed of hand gestures, thus the recognition rate was 79%.

The main difficulties of the hand gesture recognition (Andrés & Marco, 2017) with EMG using machine learning are: the noisy behavior of EMG signal, and the small number of gestures per person relative to the number of generated data by each gesture (overfitting). Whereas, in Chun-Jen et al. (2017) the training process of a deep-learning neural network required a large amount of training data, they combined a large set of computer-generated 3D hand images with few real camera images to form the training data set, the testing and training sets had 24 classes of hand gestures, and the results accuracy was 77.08%.

The authors of Deepali & Milind (2016) considered 26 different alphabets of American Sign Language, with a total of 520 samples (consisting of 20 samples of each alphabet), and results gave an accuracy of 96.15%. In study of Stefano et al. (2018) to avoid correlation between training and testing datasets the Leave One Subject Out (LOSO) cross-validation technique was used, and the accuracy was 92.87%.

## The Future of Hand Gesture Recognition

Despite the high performance of some of the recent methods discussed in this research, the hand gesture recognition is still an evolving topic that needs more experiments. Hand gesture recognition method also needs to be extended to cover all of the areas of information technology and artificial intelligence, such as tablets, smartphones, gaming consoles, smart televisions, laptops and desktops (Hexa, 2017).

It is no doubt that hand gesture recognition is capable of enabling natural communication and intelligence into applications that humans use every day. Hand gesture recognition is employing the principle of perceptual computing and changing the methods of human computer interaction (HCI) making them less complex and more enjoyable.

Applications such as home control systems, healthcare systems, gaming technologies, automobiles, televisions, home automations, and robotics are expected to be able to use hand gesture recognition to represent the communication between the user and the devices (Hexa, 2017). Furthermore, some of the applications are very sensitive and in need of having a high recognition accuracy almost close to 100% to be able to use them without causing any damage or danger to human lives; such as applications of the health field, the transportation field, and the flight operation field.

As hand gesture recognition was recently applied in some gaming consoles and has increased the sales rate of these consoles (such as Xbox and PlayStation), it is expected to keep growing more and more over time. Smartphones are expected to experience the highest growth of hand gesture recognition (Hexa, 2017). Smart televisions are also expected to experience a growth in this topic and increase the purchasing rate of the latest technology using hand gestures (Hexa, 2017). The topic is expected to grow over 28% from the year 2017 to 2024 (Hexa, 2017).

## Conclusions

This paper had successfully presented the most prominent techniques, applications, and challenges in hand gesture recognition. These include the gesture acquisition methods, the feature extraction process, the classification of hand gestures, the applications that were recently proposed in the field of sign language, robotics and other fields, the environmental surroundings challenges and the datasets challenges that face researchers in the hand gesture recognition process, and the future of hand gesture recognition.

The results of this paper can be summarized as the following; the surface electromyography (sEMG) sensors with wearable hand gesture devices were the most acquisition tools used in the works studied. Also, the Artificial Neural Network (ANN) was the most applied classifier, the most popular application was using hand gestures for sign language, the dominant environmental surrounding factor that affected the accuracy was the background color, and the common problem found in many of the studies was overfitting in the datasets.

##  Supplemental Information

10.7717/peerj-cs.218/supp-1 Supplemental Information 1 Systematic Review Dataset Data include a sample of abstracts that were scanned in this work.

10.7717/peerj-cs.218/supp-2 Supplemental Information 2 PRISMA Checklist

10.7717/peerj-cs.218/supp-3 Supplemental Information 3 Contribution that the systematic review makes to knowledge in light of previously published related reports, including other systematic reviews

10.7717/peerj-cs.218/supp-4 Supplemental Information 4 Rationale of conducting this systematic review

## Additional Information and Declarations

### Data Availability

The following information was supplied regarding data availability: The data used in the experiments of our research was collected from IEEE, and is available at Figshare: Yasen, Mais (2019): Literature.rar. figshare. Dataset. https://doi.org/10.6084/m9.figshare.8158874.v1. A sample of the data is available in the Supplemental Files.
