# Supplementary material for: A systematic review on hand gesture recognition techniques, challenges and applications
Source: PeerJ Comput Sci. 2019 Sep 16;5:e218. doi: 10.7717/peerj-cs.218 (PMC7924500; doi:10.7717/peerj-cs.218)
Supplement: Supplemental Information 1 — Data include a sample of abstracts that were scanned in this work. [file peerj-cs-05-218-s001.docx]

**Systematic literature review**

| **Reference number** | **Abstract** | **RQ** | **Not RQ** |
| --- | --- | --- | --- |
| **1** | The work in [1] applies two of methods of feature extraction, a contour hand and complex Alhzat. The classification was done using artificial neural network using the background learning algorithm. The training data had 25 images. The results reached an accuracy of 82.2%. | X |  |
| **2** | In [2] a real-time vision-based hand gesture recognition system was proposed. The system consists of RGB image acquisition module, image preprocessing module with skin color detection and binary quantization, feature extraction module using Discrete Fourier Transform (DFT) operations of histograms of vertical and horizontal, classification was applied using neural network. They trained it using 24 static American Sign Language (ASL). The results showed that their system got a high accuracy of 93.3%. | X |  |
| **3** | In experiments of Fuchang Yang and Z hijian Shi [3], they used a static hand gesture recognition method combined with depth data to enhance the performance of human computer interaction Kinect hand gesture recognition and because the vision-based hand gesture recognition requires environmental background. The use of depth data was to help in hand separation and to accomplish a synchronized color and depth images in Kinect. They used detailed features as an input to K-nearest neighbor (KNN) classifier to recognize hand gestures and to avoid having imbalanced data and images. They evaluated their method and verified the robustness using recognition rate, light and rotation, translation and scale change. The results showed that using the depth information has reduced the effect of illumination and increased the stability of the recognition. | X |  |
| **4** | As mentioned in [4] The authors used a static hand gesture recognition in a real-time human-computer interaction application to control the Power Point presentations from a distance. They did not use any traditional methods for hand gesture recognition such as hand-gloves, markers, rings, pens or any other devices. Their proposed approach takes the input data from a portable webcam then processes the images and after that it extracts a histogram of gradients features. The processed image is then compared with the database of images they have before using K-nearest neighbor algorithm (KNN). Their approach was tested using different light brightness (dull, medium, and bright). The results showed that the gesture images were detected better when the background had a brighter light and the total accuracy was (80%). | X |  |
| **5** | As showed in [5] the authors developed an alternative to talking which is sign language recognition and hand gestures. Hand gestures are represented by hand shapes, orientations and movement of the hands, alignments of the fingers, and the palm position. Their objective was to do segmentation of the hands using polygon approximation and approximate convex decomposition, then record the unique feature between the various convex segments of the hand as a method of feature extraction. They used Support Vector Machine (SVM), Artificial Neural Network, Naive Bayes and K-Nearest Neighbor (K-NN) classifiers as the training method to recognize the features extracted. The obtained results were promising with tolerable acceptance rate. | X |  |
| **6** | The authors Rosalina, Lita Yusnita, Nur Hadisukmana, R.B Wahyu, Rusdianto Roestam, Yuyu Wahyu in [6], produced a sign language that consists of the alphabetic from “A” to “Z”, the numbers from “0” to “9”, and some additional punctuation marks like “Period”, “Question Mark”, and “Space” using static hand gesture recognition. Hand gestures are obtained by evaluating the contour captured from the image segmentation using a glove worn by the speaker and then it is classified using Artificial Neural Network that was trained before. The results showed that the accuracy of hand gesture recognition obtained was 90%. | X |  |
| **7** | In the experiments of [7], the authors produced a sign language communication using hands gestures. To overcome the problem of having more than one meaning and move to represent a gesture they used vision-based hand gesture recognition systems and data glove-based hand gesture recognition systems. They implemented two different techniques of vision-based hand gesture recognition and one data glove-based technique. The vision-based techniques are static hand gesture recognition technique and real-time hand gesture recognition technique. In data glove-based technique the glove had five flex sensors, to recognize the hand gesture the change in resistance of the flex sensors was taken into consideration. The results showed that the vision-based techniques gave 100% accuracy in bright lighting condition with a white background while the data glove-based technique gave an accuracy of 86%, and the vision-based technique was more stable and reliable compared to the data glove-based technique. | X |  |
| **8** | The authors of [8] presented a wearable hand gesture recognition system, which decoded the information from surface electromyography (sEMG) and micro-inertial measurement unit μ-IMU. The number of sEMG electrodes was eliminated to two without scarifying the accuracy of recognition and shortened the distance to real applications. The authors also applied a capacitive coupled body channel communication (CC-BCC) module for wireless communication to make sure that low-power and high-security are accomplished. To recognize the patterns, they used a modified deep forest algorithm. The results showed that 16 hand gestures include 10 dynamic and 6 static gestures were correctly recognized and the accuracy was 96% in less than 6 ms. | X |  |
| **9** | In [9] a recognition frame of continuous hand gestures of upper-limb exoskeleton robot was proposed. Real time gesture recognition based on sEMG. The hand gesture was modeled and decomposed by the use of Gaussian Mixture Model-Hidden Markov Models (GMMHMM). GMMs are used as sub-states of HMMs to decode sEMG feature of gesture. | X |  |
| **10** | In [10] a hand gesture system using two antennas was proposed. They proposed to use two monopole antennas to measure signatures at different locations. They tested their system using ten hand gestures. Deep convolutional neural network was used for classification. Pre-trained networks such as AlexNet and VGG-16 that were trained for general optical images were able to maximize the classification accuracy of their hand gesture recognition problem. | X |  |
| **11** | As mentioned in [11] a barometric-pressure-sensor-based wristband was presented for hand gestures recognition. They had three groups of hand gestures: six wrist gestures, five single finger flexions, ten Chinese number gestures. support vector machine (SVM) was used for classification. Results showed classification accuracies of 98% for the wrist gestures, 95% for the single finger flexions, and 90% for Chinese number gestures. | X |  |
| **12** | As showed in [12] the authors presented a human-computer interaction based on hand gesture in a projector-camera system. However, it is difficult because of the complex backgrounds, variable external illumination, and shadows of hand gesture. regional contrast based salient object extraction algorithm (RC), and a method using the color statistics of image were used. Results showed that the proposed method had an excellent performance using Bayesian color model for skin detection (Bayes). | X |  |
| **13** | The authors of [13] proposed a real-time hand gesture recognition for flight control of multiple quadrotors through electromyography signals (EMG) and Convolutional Neural Network (CNN) for classification. Also, a Sliding Mode Control(SMC) algorithm is implemented to control the quadrotors formation during flying, based on leader-follower principle. The results after classifying for (neutral, ok, Open hand, Wrist radial deviation, Ulnar deviation, Wrist extension, Wrist flexion, Closed fist) gestures reached an accuracy of 100% for Ulnar deviation and Closed fist gestures. | X |  |
| **14** | As mentioned in [14] The authors presented a continuous hand gestures recognition technique using three-axis accelerometer and gyroscope sensors in smartphones. A gesture coding algorithm is used to reduce the influence of unstableness. To detect the start and end points of gestures a gesture spotting algorithm was applied. They used the dynamic time warping algorithm for classification. | X |  |
| **15** | In [15], an American Sign Language (ASL) system for people who are deaf or hard-of hearing was proposed based on action recognition techniques to recognize individual ASL signs. They used gestures of both hands, body movements, and facial expressions. The used RBG and depth videos of multi-sentence ASL performances for training. They followed 5 different feature extraction strategies; Depth image sequence, Body joints & Facial landmarks, Hand shapes & facial expressions/attributes. They used linear SVM for classification. The results showed good performance. | X |  |
| **16** | As presented in [16] the authors came up with two dynamic hand gesture recognition techniques using low complexity recurrent neural network (RNN) algorithms for wearable devices. The first was based on video signal and uses convolutional neural network (CNN) with RNN for classification. The other uses accelerometer data and applies RNN for classification. To optimize the memory size and reduce the power consumption a Fixed-point optimization was used. The results showed that the required memory space for weights was reduced by 6.25%, and the RNN based implementations showed good results. | X |  |
| **17** | The authors of [17] produced a control system for robotic wheelchair for the aged and the disabled. Which contained two parts: gesture interaction and intelligent wheelchair. They used the Kinect camera for gesture tracking. The segmentation algorithm used was based on skin color and depth information method. The system changes speed according to the position of the palm in real time. The results showed an accuracy of approximately 99%. | X |  |
| **18** | As mentioned in [18], the authors presented the design of a convolutional neural network architecture using the MatConvNet library in order to achieve the recognition of 2 classes: “open” and “closed” and “unknown”. Their data contained six architectures with variance in the hyperparameters and depth. Also, their approach allowed the consequent execution of a robotic agent, in example the delivery of objects. The results of neural networks gave the best performance. | X |  |
| **19** | As mentioned in [19], the authors presented the recognize of the static hand gestures with different features like: scale, rotation, translation, illumination, noise and background. They used the alphabet of sign language of Peru (LSP). Digital image processing techniques are used to eliminate noise, to improve the contrast under different illumination, to separate the hand from the background and to cut the region containing the hand. Two convolutional neural networks (CNN) architectures with different number of layers and parameters per layer were applied to classify the 24 hand gestures to improve the rate of recognition accuracy. The first CNN got an accuracy of 95.37% and the second CNN -with greater depth or number of hidden layers- got an accuracy of 96.20% and images with complex background got low accuracy. | X |  |
| **20** | The study of [20] presented a new model for hand gesture recognition in real time based on the EMG of the forearm. The input of this model is the surface electromyography measured by the commercial sensor the Myo armband placed on the forearm. The output is the label of recognizing 5 classes of gestures: pinch, fist, open, wave in, and wave out. For the classification stage, they used the k-nearest neighbor rule together with the dynamic time warping algorithm. This approach could be used in multiple applications in medical and engineering fields. The results of their model gave better accuracy (86%) than the Myo system (83%). | X |  |
| **21** | The study of [21] showed hand Gesture Recognition on top-view hand images observed by a Time of Flight (ToF) camera in a car for touchless interactions inside a car. The authors aim to improve the detection of hand-gestures by correcting the probability estimate of a Long-Short-Term Memory (LSTM) network by pose prediction performed by a Convolutional Neural Network(CNN). They used Principal Component Analysis(PCA) as a training procedure to reduce the labelled data of hand pose classification to perfect the initialization of weights for the CNN. They used five hand poses with a nine class hand gestures. The results showed that their approach got an accuracy of 89.50%. | X |  |
| **22** | The experiments of [22] illustrates a presented a technique to detect static hand gesture using both RGB and depth data. RGB cameras can be used to detect hand, but it has a limited application because hand detection can be hard in some lighting conditions or in different skin colors. Using depth camera data is better to distinguish hands in front of camera in a better way. After detecting the user’s face in RGB frame, then analyze the pixels in depth mapping to detect arms and recognize hand palm. | X |  |
| **23** | Experiments of [23] propose a technique for the recognition of Lao alphabet sign language for the deaf people. Histogram of Oriented Gradients (HOG) was used for image processing, to extract characteristics of the hand. The extracted features are then sent to the template matching process. The similarity is measured by correlation coefficient measurement technique. They used 54 Lao alphabets in the experiments. Testing data had 540 gestures from 4 individuals. It was difficult to maintain constant speed of hand gesture. The recognition rate was 79%. | X |  |
| **24** | In [24] they introduced a wearable sensor suite fusing arm motion and hand gesture recognition for operator control of UAVs. The sensor suite fuses mechanomyography (MMG) and an inertial measurement unit (IMU) to capture arm movement and hand gesture. The IMU produces world referenced orientation and acceleration data while concomitant MMG tracks muscle activation through surface vibration. The use of surface muscle vibration for gesture recognition removes the need for electrical contact with the skin. They used a convolutional neural network (CNN) system used for real-time hand gesture recognition. They tested and trained for five gestures. The results achieved 94% accuracy. | X |  |
| **25** | The authors of [25] suggested that spatial localization of the hands when it contains background, human face, and human body could be a challenging task. The authors presented a deep convolutional neural network to directly classify hand gestures in images without any segmentation. They used seven kinds of hand gestures that can command a consumer electronics device, such as mobiles phones and TVs, the results achieved an accuracy of 97.1% in the dataset with simple backgrounds and 85.3% in the dataset with complex backgrounds. | X |  |
| **26** | As showed in [26] the authors proposed a wearable device with photo-reflective sensors arranged in an array to recognize hand gestures by measuring the skin deformation of the back of the hand. Finger movements can be observed because the muscles and bones on the back of the hand are linked to the fingers. Skin deformation is measured by the distance between the device and skin with these sensors. They used a support vector machine for recognition of 20 hand gestures. The results showed that their system performed at a suitable level of accuracy. | X |  |
| **27** | The experiments of [27] presented a hand gesture recognition based on 3-channel electromyography (EMG) sensors by tracking the cross points of EMG signals and their moving average curves. They classified the recognition using k-Nearest Neighbor (kNN) and Decision Tree algorithms combination. After using 10 kinds of hand gestures, the results showed that the decision tree increases the recognition accuracy and reduces the recognition time. | X |  |
| **28** | In [28] the authors used Naive Bayes as a training method for normal people to learn sign language and communicate to people with hearing disability, to classify hand gesture in Indonesian Sign Language System (SIBI). they used a low cost and portable finger motion capture device called Leap Motion. Their data contained 10 gestures, and 19 features were used. The results showed they achieved 70.7% accuracy and they recommended to avoid sun light because it disturbs the infrared sensor of the leap motion device. | X |  |
| **29** | The authors of [29] presented a static hand gesture recognition using Kinect v2 depth sensor to capture color, and infrared and depth frames. They applied convolutional neural networks architectures to classify the gestures and evaluated the effect of kernel size on the recognition accuracy. They expanded the best architecture by redoubling the number of kernels in convolutional layers. The best results were in color images. | X |  |
| **30** | In experiments of [30] The authors proposed an Augmented Reality (AR) to coalesce the virtual world into the real world and to enhance the perception of the real world by adding 2-D virtual objects. They used a new Hand Gesture Control in Augmented Reality System (HGCARS) where the gesture recognition is performed using a secondary camera and the reality is captured using an IP camera. The virtual object is added to the video feed obtained from an IP camera and controlled by using the position and depth of hand, measured using a webcam. The user can add new gestures and assign it to different functions. | X |  |
| **31** | The experiments of [31] presents a human-machine interface using three parallel electromyographic bands around the user forearm. An artificial neural network with SoftMax output layer was used for classification. The test data had nine gestures. The result using 24-electrode configuration was 0.96, using two-band bipolar was 0.95, and using single-band chained differential was 0.94. | X |  |
| **32** | The authors of [32] studied the feasibility of an interface based on a mechanomyographic signal (MMG). A test setup consisting of 5 IMU sensors arranged in a band form was used. The classifier for 5 gestures (fist, pronation, supination, flexion, extension) and idle state was implemented by using a feedforward neural network with softmax output. The feature vector consists of 18 features: 5 representing muscle activity (RMS) and 13 parameters corresponding to relative sensor orientation. The results showed that the F score was 94±6%. | X |  |
| **33** | In [33] a hand gesture recognition system based on microwave transceiver and deep learning algorithm was presented. A Doppler radar sensor with dual receiving channels was used to acquire a big database of hand gestures signals. The received hand gesture signals are then processed with time-frequency analysis. They used a deformable deep convolutional generative adversarial network for classification. The results showed an enhancement of 10% on the recognition rate. | X |  |
| **34** | In [34] a Hand Gesture Recognition system to detect the gestures with the help of image capturing devices installed on computer and other gadgets was implemented. The Accurate End Point Identification method was implemented and applied on gesture images which are captured in varying background to detect edge points and branch points and it was also applied on blurred images containing multiple objects. The AEPI method accurately recognized the gestures from such images and provided a new dimension to implement user interface that helped to provide more natural inputs through hand gestures. The Results got an accuracy of 99%. | X |  |
| **35** | In [35] a feature extraction method for hand gesture recognition from image frames is proposed. The authors employed higher order local autocorrelation (HLAC) feature extraction method. The features are extracted using different masks from Grey-scale images for characterizing hand’s image texture with respect to the possible position, and the product of the pixels marked in white. Then features with the most useful information are selected based on mutual information quotient (MIQ). Multiple linear discriminant analysis (LDA) classifier is adopted to classify different hand gestures. The results showed that the HLAC method accuracy outperformed other common methods. | X |  |
| **36** | The paper [36] describes a method of hand gesture recognition using Principle Component Analysis (PCA) implemented in Android phone. They solved the problems such as different size of gesture image captured, different angle of gesture’s rotation and flipped gesture. The results showed that the proposed method got 93.95% accuracy. | X |  |
| **37** | As mentioned in [37] the authors proposed a gesture recognition system for human-computer interaction based on 24GHz radars. They used it for hand pushing, hand pulling, hand lifting and hand shaking. Decision tree was used to classify the gestures. The results got an accuracy of 92%. | X |  |
| **38** | In [38] the authors proposed using Wavelet Invariant Moments. The hand region is separated based on the depth information. The wavelet feature is calculated by enforcing the wavelet invariant moments of the hand region, and the distance feature is extracted by calculating the distance from fingers to hand centroid. A feature vector which is composed of wavelet invariant moments and distance feature is generated. A support vector machine classifier based on the feature vectors is used to classify these hand gestures. The results showed that their approach can achieve high accuracy. | X |  |
| **39** | In [39] the authors used a Surface electromyography (sEMG) armband that can be worn above any elbow of any arm and it can capture the bioelectric signal generated when the arm muscles move. They extract five eigenvalues in the time domain and use the BP neural network classification algorithm to realize the recognition of six gestures. The results got 93% accuracy. | X |  |
| **40** | In [40] the authors proposed a Kinect based dynamic hand gesture recognition. Geometric features such as area and centroid are then extracted from each frame of the video to capture the trajectory of the moving hand and compare it with the training gestures using Dynamic Time Warping. They used Kinect by Microsoft to provide depth map using infrared sensor to separate the hand part from the background. They found that if there was a larger object than the hand the Kinect captures, wrong results will be achieved. The results showed that their approach succeeded in locating the hand in all frames. | X |  |
| **41** | As mentioned in [41] the authors proposed a real time hand gesture recognition. Their model takes as input the surface electromyography (EMG) measured on the muscles of the forearm by the Myo armband. The k-nearest neighbor and the dynamic time warping algorithms were used for classifying the EMGs. They included a detector of muscle activity to speed the time and improve the accuracy of the recognition. They tested the model using 5 gestures. The results gave an accuracy of 89.5% and outperforms the recognition system of the Myo. | X |  |
| **42** | In [42] a real-time hand gesture recognition system was proposed using electromyography (EMG) in the field of medicine and engineering, with a higher number of gestures to recognize. The proposed model had five stages: acquisition of the EMG signals, preprocessing like rectification and filtering, feature extraction like time, frequency and time-frequency, classification like parametric and nonparametric, and post-processing. The main difficulties of the hand gesture recognition with EMG using Machine Learning are: the noisy behavior of EMG signal, and the small number of gestures per person relative to the number of generated data by each gesture (overfitting). | X |  |
| **43** | In [43] the authors presented a static hand gestures represented by two hands, using a framework that can recognize complex static hand gestures by using the wristband-based contour features (WBCFs). The authors required the user to wear a pair of black wristbands on his two hand wrists to segment the hand region. Then they extracted the WBCF of a hand gesture. Then a feature matching method was used to obtain a recognition result. They used 29 Turkish fingerspelling signs. The results showed an accuracy of 99.31% with only six training images for each gesture. | X |  |
| **44** | In [44] a data glove has developed to track the motion of the human hand using flex sensors, gyroscopes and vision data, and it was compared with Leap Motion Controller. The features used were: position, orientation, velocity and acceleration, bending angle of the fingers. Then extracted features were then sent to the virtual elastomer simulation. The results showed that the average error between Leap Motion and the Data Glove was 26.36% and 18.21%. With the finite element-based model the error was 10.13% for the Leap Motion and 33.03% for the Data Glove. Thus, the Leap Motion Controller had a high repeatability and high potential in using for Soft Finger type applications. | X |  |
| **45** | In [45], a synthetically-trained neural network was used for the 3D hand gesture identification. The training process of a deep-learning neural network typically required a large amount of training data. They combined a large set of computer-generated 3D hand images with few real camera images to form the training data set. The Testing and training sets had 24 classes of hand gestures. The accuracy was 77.08%. | X |  |
| **46** | In [46] The authors proposed using the LPSNet, an end-to-end deep neural network for hand gesture recognition with novel log path signature features. They used a robust feature, path signature (PS) and its compressed version, log path signature (LPS) to extract effective feature of hand gestures. They also presented a method based on PS and LPS to combine RGB and depth videos. They tested their approach on Sheffield Kinect Gesture (SKIG). The results an accuracy of 96.7% using RGB videos and 98.7% using RGB and Depth videos. | X |  |
| **47** | In [47] a technique for the automatic recognition of hand gestures using a 2.4-GHz continuous radar and a convolutional neural network was proposed. The neural network was trained using the images from three types of hand gesture. Another set of I–Q plot images were used for the evaluation of the recognition accuracy. The results gave an accuracy exceeding 96.6%. | X |  |
| **48** | The study of [48] presented a hand gesture-based computer mouse control system. They used skin color segmentation technique to control mouse movements, and morphological operations like structuring elements and blob counting. Various mouse operations like: cursor movements, right click and left click were tested using webcam. | X |  |
| **49** | In [49] the study presented a hand gesture recognition system with hand feature selection for low cost video acquisition device for numerical hand gesture of American Sign Language. Discrete wavelet transformation and singular value decomposition were used for features extraction. A genetic algorithm with effective fitness function was used to select optimal features by eliminating redundant and irrelevant features for improving the recognition performance. Finally, support vector machine was used to recognize the hand gestures. The results showed that the feature selection-embedded model outperforms the non-feature selection-based models. | X |  |
| **50** | In [50] the authors proposed a vision-based hand gesture recognition that includes the following steps: preprocessing of images, various feature extraction techniques and also different classification algorithms. The best classification accuracy was achieved using Euclidean distance and Eigen vector, but this result is for a very small dataset. The best result was a dataset containing nearly 720 images that uses Support vector machine for classification of images. Using Artificial Neural Network got an accuracy of 89.48%. | X |  |
| **51** | In [51] the authors presented a CMSWVHG (Control MS Windows via hand Gesture) to perform numerous windows actions using hand gestures. This application uses internal or external camera of computer for taking input with the help of OpenCV. The results showed an accuracy 82.52% and background noise affected the accuracy. | X |  |
| **52** | In [52] a mobile phone equipped with four types of sensors namely, accelerometer, gyroscope, magnetometer and orientation, was used for gesture classification. Without feature selection, the raw data from the sensor outputs are processed and sent to a Multi-Layer Perceptron classifier for recognition. The results showed an accuracy of 91.66% for single user dependent case, 87.48% for multiple user dependent case and 60% for the user independent case are obtained. | X |  |
| **53** | The work studied in [53] proposed a hand gesture recognition system using random regression forest with feature descriptors of skeletal data from a Leap Motion Controller. Their dataset was the University of Padova Microsoft Kinect and Leap Motion dataset and 24 letters of the English alphabet in American Sign Language. They did not evaluate the letters that are dynamic (like: j and z). For classification they used a random regression forest. The results showed that their approach reached an accuracy of 100% for the leap motion dataset, and an accuracy of 98.36% for the English letters dataset. | X |  |
| **54** | The authors of [54] presented a skeleton-based dynamic hand gesture recognition with a new motion feature augmented recurrent neural network. To describe finger movements finger motion features were utilized and to represent the global movement of hand skeleton the global motion features were extracted. for classification the used a bidirectional recurrent neural network (RNN) with the skeleton sequence to augment the motion features for RNN and improve the classification performance. Results show that their proposed method is effective and outperforms start-of-the-art methods. | X |  |
| **55** | In [55] a hand gestures recognition system was implemented using the incorporation of Bhattacharyya divergence into Bayesian sensing hidden Markov models (BS-HMM). Their system has two stages. The first is collecting depth images using Microsoft Kinect. The hand region is recognized by using information about the skeleton, yielding the segmented depth images. Then a histogram of the oriented normal 4D (HON4D) and a histogram of oriented gradient (HOG) are extracted to represent the motion patterns. Second, all features are transformed by combining every k consecutive feature vectors into a sequence of distributions. They compared BS-HMM to the standard HMM and the BS-HMM using the dataset of MSRGesture3D. Results showed that the proposed method outperforms the other methods. | X |  |
| **56** | In [56] the authors used wearable devices such as VR/AR helmet and glasses in a gesture recognition system, and they presented a dataset for the deep learning era named EgoGesture to train deep neural networks. This dataset contains more than 24,000 gesture and 3,000,000 frames for both color and depth modalities from 50 subjects. They tested 83 different static and dynamic gestures with 6 diverse indoor and outdoor scenes respectively with variation in background and illumination. They also tested when people perform gestures while they are walking. The results showed an accuracy ranging from 0.464 to 0.922. | X |  |
| **57** | The work presented in [57] is an automation system that can convert sign language to spoken language and help the hearing-impaired community. They used complex backgrounds with respect to skin color. The classification was done using support vector machine. Results showed a disadvantage in this system which is the lighting conditions. | X |  |
| **58** | In [58] a system for HGR that is based on dimensionality reducing the histogram of oriented gradients feature vectors by applying principal component analysis, for deaf-dumb people. Multi-class Support Vector Machine (SVM) and k-Nearest Neighbors (KNN) classifiers are used to classify the hand gestures. The results showed that the proposed algorithm achieved recognition rate of 97.69% under different hand poses and complex background with changes in lightning. Experiments showed that the accuracy with KNN classifier are better than with SVM classifier. Moreover, their system was robust to rotation, scale, translation, and lighting. | X |  |
| **59** | The work in [59] presented a dataset for hand gesture recognition. Their dataset contains about 5K labeled and 1K unlabeled single hand images. All the images are separated into 50 groups according to the types of hand gestures. For each hand gesture type, occlusion, light, shadow and background were considered. Then they trained their model using deep neural network. | X |  |
| **60** | In [60] a dynamic hand gesture recognition system was proposed. Recognition feature-set was used to model dynamic hand gestures using skeleton data. The data contained unsegmented streams of 13 hand gesture classes with either a single hand or two hands. Our approach is first evaluated on the existing DHG dataset, and then using their collected dataset. The results showed a good performance when using 14 gestures and it was the second-best result when considering 28 gestures. | X |  |
| **61** | The work of [61] a hand gesture recognition method based on salient feature point selection was proposed. They used the Kinect sensor and the hand gesture is segmented from the cluttered background. The shape feature of hand gesture is extracted from the contour, and the salient feature points were selected by a new algorithm to represent the hand gesture. They used a modified Dynamic Time Warping algorithm to classify data between two gestures. They tested three databases. The results showed that their method was adjusting to translation, rotation scaling and articulated deformation. | X |  |
| **62** | As mentioned [62] developed a wireless sensing WiCatch, a device free gesture recognition system. Data fusion-based interference elimination algorithm is proposed to diminish the interference caused by signals reected from stationary objects and the direct signal from transmitter to receiver. Then the system rebuilds the motion locus of the gesture by constructing the virtual antenna array based on signal samples. Then for classification the used support vector machines. The results showed that the WiCatch achieved a recognition accuracy over 0.96. Furthermore, they WiCatch applied on two-hand gesture recognition and got an accuracy of 0.95. | X |  |
| **63** | In [63] Convolutional Neural Networks (CNNs) was used for classification. ToF data was used which tends to be overly noisy depending on various factors such as illumination, reflection coefficient and distance. CNNs were able to extract the relevant information and learn a set of filters, using for testing ten different gestures from 20 different individuals and containing 600.000 samples overall. The results gave an accuracy up to 98.5% on validation sets comprising 20.000 data samples. | X |  |
| **64** | As mentioned in [64] a human non-verbal or gesture-based communication translation into English system was proposed. They used image processing techniques like outline investigating based on edge recognition, wavelet change, disintegration, widening, obscure disposal, commotion end, on training dataset. They also used Histogram Orientation Gradient called HOG for shape highlight extraction and most vital part investigation for list of capabilities streamlining and diminishment. They utilized diverse recordings such as day situation, family information, official information, relations. | X |  |
| **65** | The work in [65] presented a method to detect and to recognize hand gestures for generating hand gesture-based commands to control the media consumption in smart glasses. They presented a detection method that utilizes depth image obtained by incoming stereo image sequences and skin color information in a combined way. Then they presented a detected hand contours based on Bezier curve to provide an interoperable interface between a detection module and a recognition module. A set of hand gestures with a combination of open fingers and rotational angles were used for the hand gesture recognition. Results showed that the proposed method gave high a performance of detection of hand gesture. | X |  |
| **66** | In [66] the authors proposed a system for signs and gestures with computer vision system and extract sufficient number of images from it. Artificial Neural Network (ANN) was used for classification. They used back-propagated neural network and Pattern matching Techniques to make the Artificial Neural Network. The results showed that this approach of recognition decreases the error rate and increases the efficiency of the system. | X |  |
| **67** | In [67] hand gesture recognition for sign language using computer vision from real-time video stream was proposed. The proposed system identifies hand-palm in video stream based on skin color and background subtraction scheme. They also used a polygonal shape approximation strategy with a special chain-coding for shape-similarity matching. The results showed that the system successfully recognizes hand gestures corresponding with acceptable accuracy. | X |  |
| **68** | In [68] hand gesture recognition or finger angle prediction based on US imaging for Ultrasound imaging was proposed. A method of extracting forearm muscle information via multiple single-element US transducers was also proposed. Results showed an average recognition accuracy of 96% including five finger flexions and rest state. Linear discriminative analysis (LDA) was chosen for classification. | X |  |
| **69** | In [69] hand gesture recognition with two neural network methods. Recognition was for 10 hand gestures, images were captured on two different backgrounds and with several space orientations. Histogram of Oriented Gradients method was applied for feature extraction. Classification was performed with multilayer feedforward neural network with backpropagation algorithm. Both classification methods achieved accuracy of about 92.5%. | X |  |
| **70** | In [70] a framework for recognizing Sinhala sign language gestures and translating them in to natural language for hearing & speaking impaired people was proposed using a wearable gesture recognition armband MYO. A combination of gestural data (surface Electromyography sEMG) that measures the muscle activity and spatial data (accelerometer, gyroscope & orientation) that measures hand movements for the sign recognition was used. Multiple artificial neural networks were used for classification. The results showed an accuracy of 100% for personalized gestures and 94.4% for generalized gestures. | X |  |
| **71** | In [71] American Sign Language hand recognition system was proposed, for hearing and speech impaired people. The signs are captured using new digital sensor called “Leap Motion Controller”. Proposed system used Multi-Layer Perceptron (MLP) neural network with Back Propagation (BP) algorithm to build a classification model. They have considered 26 different alphabets of American Sign Language, with a total of 520 samples (consisting of 20 samples of each alphabet). Results gave an accuracy of 96.15%. | X |  |
| **72** | The work in [72] a hand gesture recognition technique on a smartphone that employs the Gaussian Mixture Models (GMM) based on human skin pixels and tracks segmented foreground using optical flow to detect hand swipe direction was proposed. Using Google Cardboard (GC) and Wearality2 in the phone. | X |  |
| **73** | In [73] The authors presented an algorithm of Hand Gesture Recognition using Dynamic Time Warping. The system consists of three modules: real time detection of face region and two hand regions, tracking the hands trajectory, gesture recognition based on analyzing variations in the hand locations along with the center of the face. Their system overcame the limitations of a glove-based approach and the vision-based approach concerning different illumination conditions, background complexity and distance from camera. Results showed an accuracy of 90% in recognizing 24 gestures based on Indian Sign Language. | X |  |
| **74** | In [74] A hand gesture technique Wearable electronics embedded with advanced sensors was proposed. An armband, utilizing raw nine-axis inertial motion signals was used. They used feedforward neural networks with backpropagation for classification. They Employed Daubechies wavelet transforms for feature extraction. Results gave an accuracy of 88%. | X |  |
| **75** | As mentioned in [75] recognition of dynamic hand gesture (digits). The method consists of three-fold novel contributions: finding the flow of hand they proposed new method to find flow of hand for special signs using chain code, secondly recognition technique of signs (Dynamic digits) that is independent of size and color of hand using binary images, and thirdly classification of gesture 11 to 20 (digits) using Principal component analysis. Results reached an accuracy of 94%. | X |  |
| **76** | In [76] a hand gesture system for Sign language for hearing and speech impaired people was proposed. Data acquisition was done using Camera interfacing, image segmentation by doing image quality enhancement and segmentation and color filtering process in RGB format using Erosion and Dilation, hand tracking, feature extraction using Blob Detection and Contour extraction and gesture recognition using SVM. The accuracy reached 0.85. | X |  |
| **77** | In [77] hand gestures recognition in human–robot interaction (HRI). They used a novel data glove called YoBu to collect data for gesture recognition. They also used extreme learning machine (ELM) for gesture recognition. | X |  |
| **78** | In [78] a real-time hand gesture recognition technique for presentation was proposed, to control OS on the projected screen for a virtual mouse system without any hardware requirement only one camera source is required. first glove tracking is done and then fingertips are detected with respect to centroid of the hand. | X |  |
| **79** | In [79] The authors used kohonen Self Organizing Maps as a type of Neural Networks to classify data sets in unsupervised manner to convert hand gestures into Filipino words. The system used a webcam to capture hand images. Image processing techniques such as: color segmentation, visual-hand tracking, pre-processing, and feature extraction were used. The results showed and accuracy of 97.6%. | X |  |
| **80** | In [80] hand gesture in sign language for non-verbal communication was used for deaf & dumb people who have hearing or speech problems. Features used were orientation, Centre of mass centroid, fingers status, thumb in positions of raised or folded fingers of hand, features were extracted using Color jiltering and skin segmentation. convexity hull algorithm was implemented just for finger point detection and number recognition. | X |  |
| **81** | In [81] Hand-gesture-based commands was proposed to replace touch and electromechanical input panels using vision-based mid-air unistroke character input. An acquisition module that spots the legitimate gesture trajectory by implementing pen-up and pen-down actions using depth thresholding and velocity tracking. The extracted trajectory is recognized using the equipolar signature (EPS) technique. it was applicable for rotation, scale, and translation variations, directions. The dataset contained digits, alphabets, and symbols. The result reached an accuracy of 96.5% The proposed scheme was also tested on an open dataset DAIR (Dataset for AIR Handwriting), and it got an accuracy of 95.5%. | X |  |
| **82** | In [82] continuous trajectory path made by hand over a period of time was considered for real time dynamic gesture recognition purpose using kinect sensor, for controlling robotic arm. Conventional method utilize separation of hand from surrounding environment and then finds of palm points. The human hand will be processed in OpenCV. Arduino will utilize the image for controlling DC servo motor action. Hidden Markov Model was used for classification. | X |  |
| **83** | In [83] Gesture Recognition system was proposed. Mudra is an expressive form of gesture that is mainly used in Indian classical dance form where the gesture is in visual form to connect with the audience. features extracted were: size, shape, color or texture. An attempt in computer aided recognition of Bharatnatya Mudras with Artificial Neural Network for classification. The results showed an accuracy of 97.8% | X |  |
| **84** | In [84] a hand gesture-based control design is proposed for mobile robots. Mobile robots can move according to the signals encoded by hand gestures. The gesture region is segmented from complicated background and the gestures are recognized by using some techniques such as image processing, image filtering processing, morphological image processing, image contour processing. Then a template matching algorithm is proposed with the help of the invariant moment matching method to recognize the hand gestures. maximum accuracy recorded was 85%. | X |  |
| **85** | In [85] a vision based hand gesture recognition system was proposed. Each hand part was modeled as a convex hull and pairwise distance between the parts was calculated using GJK-EPA algorithm. They used RGB and depth images. The results showed that their model was very accurate. | X |  |
| **86** | In [86] Hand shape recognition using Computer Vision was proposed. Principal Component Analysis (PCA) was used to reduce the dimensionality and extract features of images of the human hand. The dataset used was the alphabet of Irish Sign Language. Blurring using a Gaussian filter is applied to these images in order to reduce the non-linearity. k-Nearest-Neighbor (k-NN) was used to classify the gestures. | X |  |
| **87** | In [87] a hand gesture recognition system from acoustic measurements at wrist for the development of a wearable device was proposed. A prototype with 5 microphone sensors on human wrist was used. 36 gestures in American Sign Language (ASL) were studied including 26 ASL alphabets and 10 ASL numbers. Ten features were extracted. Support Vector Machine (SVM), Decision Tree (DT), K-Nearest Neighbors (kNN), and Linear Discriminant Analysis (LDA) were compared in classification performance. Results showed that LDA offered the highest accuracy above 80%. | X |  |
| **88** | In [88] hand gesture recognition for American Sign Language (ASL) for the Deaf community was proposed using a webcam. We provide two different translation paradigms; English characters (alphabet) and complete words or phrases. The hand gesture image is processed by combining image segmentation and edge detection to extract morphological information. frames were processed using the multi-modality technique used for processing individual characters. The gesture recognition was applied using a cross-correlation coefficient-based scheme. | X |  |
| **89** | As mentioned in [89] The authors presented an integrated neural regularization method to reduce the overfitting, in fully-connected neural networks that jointly combines the cutting edge of regularization techniques; Dropout and DropConnect. feed-forward networks showed low prediction performance when data size was small. The CIFAR-10 and one-hand gesture datasets were used. Results showed that the integrated method improved classification performance. | X |  |
| **90** | The work presented in [90] deals with a robotic arm using hand gestures for many applications such as automation, medical and gaming. to improve recognition rate of hand gestures, various image processing techniques are to be used such as Histogram Equalization, Median filtering, Average filtering, and Morphological filtering. after feature extraction image matching was done using cross-correlation co-efficient. | X |  |
| **91** | In [91] The authors presented a real-time hand gesture recognition by using Kinect sensor, to control mouse by user hands for operations such as 'clicking', 'dragging' and 'dropping', and engaged/disengaged gestures. | X |  |
| **92** | In [92] Gesture Recognition was used for Bio Robotics. The paper focused on presenting a sensor based human gesture recognition for the Hand Cricket game. Both the players wear the Myo armband. Myo armband is used to capture the Bio-potentials during every muscle action. Machine Learning techniques are performed to classify all the five different gestures with maximum accuracy. Support Vector Machine (SVM) gave the maximum accuracy with an accuracy of 92% and 84% for both players. | X |  |
| **93** | In [93] Indian Sign Language (ISL) hand gesture recognition system was introduced. The proposed techniques relied on multiple representations namely HOG, GIST and BSIF. They used feature fusion which is the process of combining two feature vectors to obtain a single feature vector. A random forest classifier was used. The results gave an accuracy of 92.20%. | X |  |
| **94** | In [94] The authors presented a hand movement recognition algorithm based on Average Threshold Crossing (ATC) technique. It exploits the number of threshold crossing events from the surface Electro Myo Graphic (sEMG) signals of three forearm muscles to detect four different movements of the wrist: flexion, extension, abduction and grasp. A Support Vector Machine (SVM) model was used for classification. To avoid correlation between training and testing dataset the Leave One Subject Out (LOSO) cross-validation technique was used. The ATC accuracy was 92.87 % which means it is suited for wearable systems or Internet-of-Things (IoT) applications. | X |  |
| **95** | In [95] a method for hand gesture recognition using convexity defect and background subtraction was used. The background subtraction is used to eliminate the useless information. Images processing techniques were applied to find contour of segmented hand images. Then the convex hull and convexity defects for this contour was calculated. The features must be able to characterize gestures, and invariant under translation and rotation of hand gesture to ensure reliable recognition. To extract a series of features based on convex defect detection a model was proposed, taking advantage of the close relationship of convex defect and fingertips. Five hand gestures classes were tested (fingers). | X |  |
| **96** | The gesture-based human-computer interface requires new user authentication technique because it does not have traditional input devices like keyboard and mouse. In this paper, we propose a new finger-gesture-based authentication method, where the in-air-handwriting of each user is captured by wearable inertial sensors. Our approach is featured with the utilization of both the content and the writing convention, which are proven to be essential for the user identification problem by the experiments. A support vector machine (SVM) classifier is built based on the features extracted from the hand motion signals. To quantitatively benchmark the proposed framework, we build a prototype system with a custom data glove device. The experiment result shows our system achieve a 0.1% equal error rate (EER) on a dataset containing 200 accounts that are created by 116 users. Compared to the existing gesture-based biometric authentication systems, the proposed method delivers a significant performance improvement. |  | X |
| **97** | Abstract—This paper describes a global framework that  enables contactless human machine interaction using computer  vision and machine learning techniques. The main  originality of our framework is that only a very simple  image acquisition device, as a computer camera, is sufficient  to establish a rich human machine interaction as traditional  devices such as mouse or keyboard. This framework is based  on well known computer vision techniques and efficient  machine learning techniques are used to detect and track  user hand gestures so the end user can control his computer  using virtual interfaces with very simple gestures. |  | X |
| **98** | Abstract—State-of-the-art techniques for eating activities  analysis in dietary monitoring require significant user intervention,  which is reported to be one of the major reasons for  low adherence. There are limited works using wearables for  fine-grained analysis of eating activities in terms of the eating  speed, the type of food consumed, and the portion sizes. In  this paper, we propose FIT-EVE&ADAM, an armband based  diet monitoring system that provides such fine-grained analysis,  triggered by a single hand gesture. The system collects the  user’s gesture using sensors such as electromyogram embedded  in the armband device, along with food image data using color  and thermal cameras. Finally, a novel feature selection method  is applied on the data features to estimate eating speed and  caloric intake with high accuracy (0.96 F1 score). |  | X |
| **99** | Abstract—Virtual Musical Instrument is an interface  allowing performers to simulate the instrument either in air or  by running the console application. The user keeps playing the  virtual instrument as it is real one and visually sense for  appropriate music. In this paper an attempt is made to give a  brief overview of human interaction with systems for playing  Indian musical instruments virtually. The system has to take  the hand gestures as input to the musical interface and with the  help of signal or image processing techniques like  preprocessing, segmentation, feature extraction and mapping  along with suitable machine learning algorithms, should be  able to produce the actual output of the physical instrument to  be played and handle sound related aspects in terms of volume,  tone, pitch, rhythm etc. Sensors supporting to track the hand  finger’s 3D parameters are identified and these features acting  as an input to a digital musical interface with the help of  appropriate gesture recognizing algorithms matching musical  note of the actual physical instrument. Accurately recognizing  3D gestures poses different research challenges. Authors  present an overview of latest sensors with different methods  for finger motion capturing with its features, advantages and  limitations |  | X |
| **100** | Abstract—Advances in inertial sensors technology used in  smartwatches make it possible for movement-based therapies  to be both therapeutic and evaluative at the same time.  Therapeutic Instrumental Music Performance (TIMP) uses  instrument playing for motor rehabilitation. However, applications  of TIMP are not generalizable to a variety of motor  disabilities and do not often consider the benefits of social  interaction. To address the missing opportunities and potential  strengths of an integrated technological and therapeutic  application, we introduce MIMES (Motion Initiated Music  Ensemble with Sensors). MIMES uses commercially available  smartwatches and hand gesture recognition techniques to  trigger instrumental sounds with simple hand gestures and  support multiple users. We developed this system to provide  rehabilitation programs and music therapy for a variety  of individuals with motor impairments to improve both  physical and mental health outcomes. We discuss MIMES  system architecture, gesture recognition algorithm, future  experimental design and a self-evaluation module to test the  efficacy of the technology for rehabilitation. |  | X |
| **101** | Abstract—The increasing usage of wearable devices for ambulatory  monitoring and pervasive computing systems has given  rise to the need of convenient and efficient activity recognition  techniques. Hand-dominated activity recognition has great  potential in understanding users’ gesture and providing contextaware  computing services. This paper investigates the feasibility  and applicability on the usage of wristband-interaction behavior  for recognizing hand-dominated activities, with the advantage of  great compliance and long wearing time. For each action, sensor  data from wristband are analyzed to obtain kinematic sequences.  The sequences are then depicted by statistics-, frequency-, and  wavelet-domain features for providing accurate and fine-grained  characterization of hand-dominated actions, and the correlation  between the wristband-sensor features and the actions is analyzed.  Classification techniques (Naive Bayes, nearest neighbor,  neural network, support vector machine, and Random Forest)  are applied to the feature space for performing hand-dominated  activity recognition. Analyses are conducted using the data from  51 participants with a diversity in gender, age, weight, and height.  Extensive experiments demonstrate the efficacy of the proposed  approach, achieving a recognition rate of 97.29% and an Fscore  above 0.94. Additional experiments on the effect of feature  selection and wristband sampling rate are provided to further  examine the effectiveness of our approach. Our data are publicly  available. |  | X |
| **102** | Abstract—This research proposes an interactive threedimensional  (3-D) application which can be used for modelling  and manipulating interior architectural environments. This  incorporates the concept of Virtual Reality [1] (VR) giving the  user a real world experience. Virtual Reality is a trending  technology which is gaining popularity due to the user experience  it gives and its effectiveness. The proposed system consists of 4  major components. The user can add objects to the environment  either using a description given in natural language or by giving  a hand drawn sketch of the objects to be added. Natural Language  Processing (NLP) techniques will be used to extract object details  from the user description and Image Processing techniques will  be used to extract object details from the hand drawn sketch. The  user can control the 3D environment either by using hand  gestures or voice commands. The hand gestures will be captured  using a web camera and the voice commands will be captured  using a microphone and Natural Language Processing (NLP)  techniques will be used in order to extract the user’s command.  The 3-D application will be developed using VRML [2] (Virtual  Reality Markup Language) which is a language for describing  three-dimensional (3-D) image sequences and possible user  interactions to go with them. Using the application developed  using VRML, the user can interact with the virtual environment  by viewing, navigation, moving and rotating objects within the  environment thus |  | X |
| **103** | Abstract— This research develops a Gesture based  technique to manipulate a ABB’s industrial robot IRB1520ID  using Microsoft’s Kinect Sensor. Kinect is used for acquiring  skeletal data and coordinates of each joint of a person and send  live frames to MATLAB for processing. Our MATLAB code will  calculate joint angles and send signals to the robot. The robot’s  controller will accordingly trigger routines to move the endeffector  in the desired direction, for example if the hand is  moving towards right, the robot will also move towards right and  vice versa. Communication between MATLAB and the robot’s  controller is accomplished by using an Arduino Nano which  generates the required I/O signals understood by the robot. This  technique is very effective since it takes real time motion of the  hand and connects it to motion of the robot. |  | X |
| **104** | Abstract—The sign language recognition is the most popular  research area involving computer vision, pattern recognition  and image processing. It enhances communication capabilities  of the mute person. In this paper, we present very important  different techniques for segmenting object based key frame  selections of hand region for gesture reorganization.  We proposed video object plane generation segmentation  and histogram based segmentation these two techniques for  segmenting hand region from static image or video sequences  of single handed American Sign Language. Experimental  results demonstrate the effectiveness of our proposed scheme  for segmenting American Sign Language. |  | X |
| **105** | Abstract— Gesture recognition is an easy and hassle free  mode of human computer interaction. This paper analyzes and  implements four techniques which are distance invariant pixel  count algorithm, peak counting algorithm, objects counting  algorithm and template matching. The implementation of these  techniques in this paper is limited to finding the finger count in  moderate to complex backgrounds. Some modifications are  proposed which make the algorithms invariant to rotation,  translation and scale of hand. These techniques are shown to  work fairly well in cluttered and noisy backgrounds. They are  demonstrated on real imagery and the results are compared on  the basis robustness, accuracy and background in-variance. |  | X |
| **106** | Abstract—In view of human body motion recognition  technology, a gesture recognition method based on  LabVIEW is proposed, which is applied to the control  of medical service robot. The 3D somatosensory  camera of Kinect is used to track the human skeleton  points, and then capture human actions in real time on  the LabVIEW platform, identify different actions of  the body. Finally, the motion commands are sent to the  robot through the Bluetooth communication to  complete the robot forward, turning and wheelchair  mode conversion,etc. After testing, the method of hand  gesture recognition based on the LabVIEW, can be  very good for tracking the human body and  identifying the body's actions. The human computer  interaction of the medical service robot is realized,  which provides convenience for the assistant doctor to  complete the rehabilitation and nursing of the patients. |  | X |
| **107** | Abstract— This paper describes an indigenously  developed hands-free wheel chair for physically disabled  persons. The proposed device works based on the Head  Gesture Recognition technique using Acceleration  sensor. Conventional electric powered wheel chairs are  usually controlled by joysticks or hand gesture  technology which cannot fulfil the needs of an almost  completely disabled person who has restricted limb  movements and can hardly move or turn his head only.  Acceleration sensor is used for the head gesture  recognition and RF (radio frequency) module is used for  the smart wireless controlling. With the change of head  gesture, data is sent wirelessly to the microcontroller  based motor driving circuit to control the movement of  the Wheel Chair in five different modes, namely FRONT,  BACK, RIGHT, LEFT and a special locking system to  STAND still at some place. The proposed device is  fabricated using components collected from local market  and tested in lab for successful functioning, test results  are included in this paper. |  | X |
| **108** | Springs have been employed in a wide range of mechanical systems. This work deals with the design of multi degree-of-freedom spring mechanism. The adaptable spring is desired for enhancing performances of various mechanical systems employing springs. We demonstrate that such adaptable springs can be realized by adopting anthropomorphic musculoskeletal structures of the human upper-extremity, which possesses highly nonlinear kinematic-coupling among redundant muscles existing in its structures. We propose multi-degree-of-freedom spring mechanisms resembling the musculoskeletal structure of the human upper-extremity, and verify the applicability of these mechanisms through simulation. |  | X |
| **109** | ABSTRACT We propose novel multi-order statistical descriptors which can be used for high speed object  classi_cation or face recognition from videos or image sets. We represent each gallery set with a global  second-order statistic which captures correlated global variations in all feature directions as well as the  common set structure. A lightweight descriptor is then constructed by ef_ciently compacting the secondorder  statistic using Cholesky decomposition. We then enrich the descriptor with the _rst-order statistic  of the gallery set to further enhance the representation power. By projecting the descriptor into a lowdimensional  discriminant subspace, we obtain further dimensionality reduction, while the discrimination  power of the proposed representation is still preserved. Therefore, our method represents a complex image  set by a single descriptor having signi_cantly reduced dimensionality. We apply the proposed algorithm  on image set and video-based face and periocular biometric identi_cation, object category recognition, and  hand gesture recognition. Experiments on six benchmark data sets validate that the proposed method achieves  signi_cantly better classi_cation accuracy with lower computational complexity than the existing techniques.  The proposed compact representations can be used for real-time object classi_cation and face recognition  in videos. |  | X |
| **110** | Abstract—In our daily life, we, human beings use our hands  in various ways for most of our day-to-day activities. Tracking  the position, orientation, and articulation of human hands has  a variety of applications including gesture recognition, robotics,  medicine and health care, design and manufacturing, and art  and entertainment across multiple domains. Out of the various  tracking methods, vision-based tracking is an efficient and widely  used method. Several devices have been developed by researchers  and engineers to track objects using vision. The leap motion (LM)  controller is one such device. However, visual tracking is an  equally complex and challenging task due to several factors  like higher dimensional data from hand motion, higher speed  of operation, and self-occlusion. This paper puts forth a novel  method for tracking the fingertips of human hand using two  distinct sensors and combining their data by sensor fusion  technique. The proposed method is tested using standard human  hand gestures, and the results are discussed. Finally, a soft robotic  gripper was operated remotely based on LM hand tracking and  the proposed sensor fusion method. |  | X |
| **111** | Abstract—Current video services are still controlled in an  old-fashioned way using keyboard and mouse for computers  and remote control for TV sets. This paper presents more  attractive and intuitive interaction methods using commercially  available motion sensing input devices. These devices are typically  based on a webcam-style add-on peripheral, thereby enabling  facial recognition, gesture control, and speech recognition. These  technologies are applied in this research to automatically authenticate  a user, enable video control (play, pause, seeking), and  browsing, selecting, and rating content by hand gestures or voice  commands. By monitoring the user’s gaze and using emotion  recognition techniques, the user’s interests and engagement with  the content can be estimated. This is interpreted as implicit user  feedback for the video content, and establishes an automatic  feedback channel, which can be used for content personalization  and recommendation. User tests showed an accurate recognition  of voice and gestures and confirmed the attractiveness and  intuitiveness of these techniques for end-users. |  | X |
| **112** | Abstract—Digital cameras are widely used in desktop and  notebook PCs. Taking self-portraits is one of the important  function of such cameras, which allows users to capture  memories, create art, and improve photography techniques. A  desktop environment with a large display and a pan-and-tilt  camera provides users with a good area for exploring more angles  and postures while taking self-portraits. However, most of the  existing camera interfaces of this type are limited to device-based  systems (i.e., mouse and keyboard) that prevent users from  efficiently controlling the camera while taking self-portraits. This  study proposes a vision-based system equipped with a gesture  interface that control a pan-and-tilt camera for taking selfportraits.  This interface uses gestures, particularly slight hand  movements (i.e., sweeps, circles, and waves), to control the pan,  tilt, and shutter functions of the camera. The gesture-recognition  achieved good efficiency in performance (less than 2ms) and the  recognition rate (0.9 on average in lighting conditions range 100 -  200). Experimental results indicate that the proposed system  effectively controls the options in a self-portrait camera, this  approach provides significantly higher satisfaction, particularly in  terms of the intuitive motion gestures, freedom, and enjoyment,  than when using a hand-held remote control or a conventional  mouse-based interface. The proposed system is a promising  technique for taking self-portraits in a desktop environment. |  | X |
| **113** | Although different interaction modalities have been proposed in  the field of human-computer interface (HCI), only a few of these  techniques could reach the end users because of scalability and  usability issues. Given the popularity and the growing number of  IoT devices, selecting one out of many devices becomes a hurdle  in a typical smarthome environment. Therefore, an easy-to-learn,  scalable, and non-intrusive interaction modality has to be explored.  In this paper, we propose a pointing approach to interact with  devices, as pointing is arguably a natural way for device selection.  We introduce SeleCon for device selection and control which uses  an ultra-wideband (UWB) equipped smartwatch. To interact with  a device in our system, people can point to the device to select  it then draw a hand gesture in the air to specify a control action.  To this end, SeleCon employs inertial sensors for pointing gesture  detection and a UWB transceiver for identifying the selected device  from ranging measurements. Furthermore, SeleCon supports an  alphabet of gestures that can be used for controlling the selected  devices. We performed our experiment in a 9m-by-10m lab space  with eight deployed devices. The results demonstrate that SeleCon  can achieve 84.5% accuracy for device selection and 97% accuracy  for hand gesture recognition. We also show that SeleCon is power  efficient to sustain daily use by turning off the UWB transceiver,  when a user’s wrist is stationary. |  | X |
| **114** | We present two case studies of motion tracking applicable to  gesture recognition in fencing and kanji sports. Through them,  we illustrate how the two basic techniques for gesture extraction  - camera based video analysis and wearable device motion  tracking - could be employed in sports applications. In the first  case, advanced video frame analysis is applied to gesture  recognition for improved fencing training. The second case  uses portable devices for tracking of hand motion and recognition  of strokes in a kanji sports educational game. Prototype  applications for both gesture recognition approaches have been  implemented on different platforms and employed in practice. |  | X |
| **115** | Abstract—Gesture recognition is highly challenging in the field  of automotive Infotainment for system control. In this paper the  technique proposed begins by detecting and tracking the rotation  of the hand trajectory and determining the variations in the hand  locations and finally recognizing the gesture movement. The  proposed method detects the rotational gesture movement by  determining the angle of the object with respect to three sensors  arranged in the form of an equilateral triangle. The rotational  gesture is determined with respect to 360 degree rotations where  the angle is determined with respect to 60 samples. It is that the  resulting information of angle measurement can detect the  direction of the gesture movement using three optical sensors to  be used in Automotive Infotainment like media playback etc. |  | X |
| **116** | Elder adults may have some dependence on performing common activities like zapping on the television through a remote control (i.e. due to possible hand mobility problems). The Internet of Things (IoT), including the Radio Frequency Identification (RFID), interconnects devices to provide a higher variety of services. Together, and by applying intelligence through Machine Learning (ML) techniques, advanced applications can be implemented improving people's life. We present the Smart Surface system, relying on state of the art RFID equipment. It uses the unsupervised machine learning technique K-means clustering to detect and trigger actions by means of simple gestures, in real time and in a non-intrusive way. We implemented and evaluated a prototype of the Smart Surface system achieving an accuracy of 100% gesture recognition. |  | X |
| **117** | Abstract—Short-range continuous-wave Doppler radar  sensors have been mainly used for noncontact detection of various  motions. In this paper, we investigate the feasibility to implement  the function of a remote mouse, an input device of a computer,  by recognizing human gestures based on a dual-channel  Doppler radar sensor. Direct conversion architecture, symmetric  subcarrier modulation, and bandpass sampling techniques are  used to obtain a cost-effective solution. An arcsine algorithm  and a motion imaging algorithm are proposed to linearly  reconstruct the hand and finger motions in a 2-D plane from the  demodulated Doppler phase shifts. Experimental results verified  the effectiveness of the proposed architecture and algorithms.  Different from the frequency-domain “micro-Doppler” approach,  the proposed remote gesture recognition based on linear motion  reconstruction is able to recognize definitive signatures for  the corresponding motions, exhibiting promising potential in  practical applications of human–computer interaction. |  | X |
| **118** | Abstract— Selection and use of pattern recognition  algorithms is application dependent. In this work, we explored  the use of several ensembles of weak classifiers to classify  signals captured from a wearable sensor system to detect food  intake based on chewing. Three sensor signals (Piezoelectric  sensor, accelerometer, and hand to mouth gesture) were  collected from 12 subjects in free-living conditions for 24 hrs.  Sensor signals were divided into 10 seconds epochs and for each  epoch combination of time and frequency domain features were  computed. In this work, we present a comparison of three  different ensemble techniques: boosting (AdaBoost), bootstrap  aggregation (bagging) and stacking, each trained with 3  different weak classifiers (Decision Trees, Linear Discriminant  Analysis (LDA) and Logistic Regression). Type of feature  normalization used can also impact the classification results.  For each ensemble method, three feature normalization  techniques: (no-normalization, z-score normalization, and minmax  normalization) were tested. A 12 fold cross-validation  scheme was used to evaluate the performance of each model  where the performance was evaluated in terms of precision,  recall, and accuracy. Best results achieved here show an  improvement of about 4% over our previous algorithms. |  | X |
| **119** | Abstract􀀀The objective of this paper has been the  development of a prototype of articulated robotic arm and the  implementation of a control strategy through gesture recognition  (Leap Motion Sensor), by means the natural movement of the  forearm and hand. The series of advances relative to the control  techniques have caused that the robotics it has also introduced as  an educational complement in obligatory basic teachings. Final  Year Project TFG is an academic task that allows to evaluate the  skills and competences acquired by students along their  university period. So students, during their development, can  implement numerous theoretical bases of an entertaining and fun  form. To develop and to control robotic elements locally or  remotely, it has always proven to be a clear example of additional  motivation on the students. The prototype developed has  exceeded the initial expectations and at low cost. |  | X |
| **120** | Abstract—The integration of augmented reality (AR) techniques  in user interface design has enhanced interactive experiences  in teleoperation of robots, hands-on learning in  classrooms, laboratory, and special education, and user training  in an array of fields, e.g., aerospace, automotive, construction,  manufacturing, medical, etc. However, AR-based user interfaces  that command machines and tools have not been fully explored  for their potential to enhance interactive learning of engineering  concepts in the laboratory. This paper outlines the development  of a mobile application executing on a tablet device, which  renders an immersive AR-based graphical user interface to  enable users to monitor, interact with, and control a fourlink  underactuated planar robot. Computer vision routines  are used to extract real-time, vision-based measurements of  the robot’s joint angles and end effector location from the  live video captured by the rear-facing camera on the tablet.  The obtained measurements are used to render AR content  to offer users with additional visual feedback. Touch gesture  recognition is implemented to allow users to naturally and  intuitively command the robot by tapping and dragging their  fingers at desired locations on the tablet screen. Experimental  results show the performance and efficacy of the proposed  system as it is operated in two different modes: one in which  the user has direct control over the angles of the actuated links  of the robot and one in which the user has direct control over  the end effector location. |  | X |
| **121** | Abstract—This paper describes a novel method called Deep Dynamic Neural Networks (DDNN) for multimodal gesture recognition.  A semi-supervised hierarchical dynamic framework based on a Hidden Markov Model (HMM) is proposed for simultaneous gesture  segmentation and recognition where skeleton joint information, depth and RGB images, are the multimodal input observations. Unlike  most traditional approaches that rely on the construction of complex handcrafted features, our approach learns high-level spatiotemporal  representations using deep neural networks suited to the input modality: a Gaussian-Bernouilli Deep Belief Network (DBN) to  handle skeletal dynamics, and a 3D Convolutional Neural Network (3DCNN) to manage and fuse batches of depth and RGB images.  This is achieved through the modeling and learning of the emission probabilities of the HMM required to infer the gesture sequence.  This purely data driven approach achieves a Jaccard index score of 0.81 in the ChaLearn LAP gesture spotting challenge. The  performance is on par with a variety of state-of-the-art hand-tuned feature-based approaches and other learning-based methods,  therefore opening the door to the use of deep learning techniques in order to further explore multimodal time series data. |  | X |
| **122** | Abstract—We present a new gesture recognition method that is based on the conditional random field (CRF) model using multiple  feature matching. Our approach solves the labeling problem, determining gesture categories and their temporal ranges at the same  time. A generative probabilistic model is formalized and probability densities are nonparametrically estimated by matching input  features with a training dataset. In addition to the conventional skeletal joint-based features, the appearance information near the active  hand in an RGB image is exploited to capture the detailed motion of fingers. The estimated likelihood function is then used as the unary  term for our CRF model. The smoothness term is also incorporated to enforce the temporal coherence of our solution. Frame-wise  recognition results can then be obtained by applying an efficient dynamic programming technique. To estimate the parameters of the  proposed CRF model, we incorporate the structured support vector machine (SSVM) framework that can perform efficient structured  learning by using large-scale datasets. Experimental results demonstrate that our method provides effective gesture recognition results  for challenging real gesture datasets. By scoring 0.8563 in the mean Jaccard index, our method has obtained the state-of-the-art  results for the gesture recognition track of the 2014 ChaLearn Looking at People (LAP) Challenge. |  | X |
| **123** | Abstract—Surface electromyography (sEMG)-based pattern  recognition studies have been widely used to improve the classification  accuracy of upper limb gestures. Information extracted  from multiple sensors of the sEMG recording sites can be used as  inputs to control powered upper limb prostheses. However, usage  of multiple EMG sensors on the prosthetic hand is not practical  and makes it difficult for amputees due to electrode shift/movement,  and often amputees feel discomfort in wearing sEMG sensor  array. Instead, using fewer numbers of sensors would greatly  improve the controllability of prosthetic devices and it would  add dexterity and flexibility in their operation. In this paper, we  propose a novel myoelectric control technique for identification of  various gestures using the minimum number of sensors based on  independent component analysis (ICA) and Icasso clustering. The  proposed method is a model-based approach where a combination  of source separation and Icasso clustering was utilized to improve  the classification performance of independent finger movements  for transradial amputee subjects. Two sEMG sensor combinations  were investigated based on the muscle morphology and Icasso  clustering and compared to Sequential Forward Selection (SFS)  and greedy search algorithm. The performance of the proposed  method has been validated with five transradial amputees, which  reports a higher classification accuracy ( 95%). The outcome of  this study encourages possible extension of the proposed approach  to real time prosthetic applications. |  | X |
| **124** | Abstract—Recent advances in smart sensor technology and  computer vision techniques have made the tracking of unmarked  human hand and finger movements possible with high accuracy  and at sampling rates of over 120 Hz. However, these new sensors  also present challenges for real-time gesture recognition due to the  frequent occlusion of fingers by other parts of the hand.We present  a novel multisensor technique that improves the pose estimation  accuracy during real-time computer vision gesture recognition. A  classifier is trained offline, using a premeasured artificial hand, to  learn which hand positions and orientations are likely to be associated  with higher pose estimation error. During run-time, our algorithm  uses the prebuilt classifier to select the best sensor-generated  skeletal pose at each time step, which leads to a fused sequence of  optimal poses over time. The artificial hand used to establish the  ground truth is configured in a number of commonly used hand  poses such as pinches and taps. Experimental results demonstrate  that this new technique can reduce total pose estimation error  by over 30% compared with using a single sensor, while still  maintaining real-time performance. Our evaluations also demonstrate  that our approach significantly outperforms many other  alternative approaches such as weighted averaging of hand poses.  An analysis of our classifier performance shows that the offline  training time is insignificant, and our configuration achieves about  90.8% optimality for the dataset used. Our method effectively  increases the robustness of touchless display interactions, especially  in high-occlusion situations by analyzing skeletal poses from  multiple views. |  | X |
| **125** | Abstract—We present a method for gesture detection and localisation based on multi-scale and multi-modal deep learning. Each visual modality captures spatial information at a particular spatial scale (such as motion of the upper body or a hand), and the whole  system operates at three temporal scales. Key to our technique is a training strategy which exploits: i) careful initialization of individual modalities; and ii) gradual fusion involving random dropping of separate channels (dubbed ModDrop) for learning cross-modality correlations while preserving uniqueness of each modality-specific representation.We present experiments on the ChaLearn 2014 Looking at People Challenge gesture recognition track, in which we placed first out of 17 teams. Fusing multiple modalities at several spatial and temporal scales leads to a significant increase in recognition rates, allowing the model to compensate for errors of the individual classifiers as well as noise in the separate channels. Futhermore, the proposed ModDrop training technique ensures robustness of the classifier to missing signals in one or several channels to produce meaningful predictions from any number of available modalities. In addition, we demonstrate the applicability of the proposed fusion scheme to modalities of arbitrary nature by experiments on the same dataset augmented with audio. |  | X |
| **126** | In the past years, the recognition of gesture feature has been glamoured attention as a natural human. The communication system can build the human relationships. The mode of communication will be verbal and non-verbal. The non-verbal communication is not only used for the physically challenged person, but also used in gaming, surveying, etc. There is no need of peripheral device to interact with the computer. In this paper the various techniques are discussed to recognize the hand gesture. In today's era, the Kinect depth data is the famous research for the identification of new fingers and the recognition of hand gesture. Finally, discuss the recognition of hand gesture will applicable in many fields. |  | X |
| **127** | In this paper, we describe an accurate, low power (<10mW), and real-time (<33.3ms) 3D HGR processor for smart mobile devices with 3 key features: 1) a pipelined CNN processing element (PE) with a shift MAC operation for high throughput by maximizing core utilization; 2) triple ping-pong buffers with workload balancing for fast line streaming by reducing external accesses; and 3) nearest-neighbor searching (NNS) processing-in-memory (PIM) for high energy efficiency by reducing the number of bitlines requiring pre-charge in SRAM. |  | X |
| **128** | Gestures are gaining significant demand in embedded devices to provide enhanced user experience through natural communication. This paper presents a robust approach of Hand Gesture Recognition to detect gestures in real time without the need of a delimiter. The method represents the gestures with trajectory strings and matches them with stored trajectory strings of pre-defined gestures. The proposed method employs an enhanced matching scheme using a custom edit distance metric that enables finding of the closest gesture in the database with speed invariance, position invariance and length invariance without the need of a delimiter. |  | X |
| **129** | In the modern world everyone should be using computers. So lot of security issues has been generated. Some hackers hack our personal details without our knowledge and it creates more problems for us. The hackers mostly hack when the system is on, then only they can able to hack that person’s details without his/her knowledge. In this project, there is a solution to overcome such security issues while providing the Numerical Pattern in security devices with help of Gesture Technology to the CPU. This project involves using four different kinds of hardware components, they are: Arduino ATMEGA328, GSMSIM900, RELAY12 volt, IR pare sensor. To control the CPU power, Turn on and Turn off while using the devices. |  | X |
| **130** | Several new interfaces provide possibilities for the realization of human-computer interaction including the controlling systems based on human movement. The human –  computer interaction is able to detect the cognitive actions, the human and computer can have much more than an interaction (HCI). Here the Human and the system form a combined capability to work together. The action of a human makes sense under Cognitive Infocommunications. In the article the mouse cursor control based on hand gestures, realized by The Leap Motion device, is introduced, as well as the evaluation of the mouse cursor movement. During the mouse cursor movement analysis, the realized movements, carried out by a conventional mouse cursor, and hand gesture detection, are compared, with the involvement of test subjects. On the basis of the results of the mouse cursor movement analysis differences were shown in the course of controlling performed by a conventional mouse, and hand gesture detection. |  | X |
| **131** | This paper describes the design and implementation of a robot that moves by hand gestures. Therefore, it removes the hassle of using joysticks or switches. The robot’s speed is also controlled during its movement by the hand. An accelerometer has been used that converts different hand gestures into data signals. The robot has two sections: the transmitting end and the receiving end. The transmitting end transmits the gesture signals and the receiving end receives them. The accelerometer works as the sensor as it senses the different gestures made by the hand and converts them into analog signals which are further processed and transmitted to the receiving end. |  | X |
| **132** | We propose a mind map manipulation system, named MMVR. This system allows multiple users to edit a mind map by using hand gestures and to share it in virtual reality space. To implement the system, we leveraged a hand gesture tracker for quick, easy, and correct handling of mind map elements and a head-mounted display (HMD) for providing a wider  workspace than a standard PC’s screen. We conducted experiments to compare our system with conventional desktop-based software under single- and multi-user conditions. |  | X |
| **133** | A large number of impairments and disabilities in human body is increasing day by day. So to improve the quality of life of disabled people researchers think on the necessity of simple and natural human-machine control interface. For the human machine interface various biomedical signals acquired from a specialized tissue, organ or cell system like the nervous system. Bio signal like EMG signal is used in various applications including identifying neuromuscular diseases, control signal for prosthetic devices, controlling machines, robots etc. EMG based hand gesture can help to develop good human-machine interface. This present an embedded solution for real-time EMG based hand gesture recognition. It involves acquisition of EMG signal, hand gesture recognition and controlling. EMG based hand gesture recognition is easy to build and a low cost system. |  | X |
| **134** | depth cameras that are located diagonally for 2 displays (front and table displays) for detecting depth and color. We propose novel method of hand gestures calibration called ‘3D hand gestures calibration method’ for simple and timesaving setup. This method can be completely applied at two displays setup with diagonal direction between depth camera and virtual space. Developers with less experience with depth camera calibration  can use proposed method easily. |  | X |
| **135** | This paper presents a method for radial shift estimation of an electrode array located around the forearm. The algorithm is aimed at band-shaped EMG human-achine interfaces recognising hand gestures. Proposed algorithm relies on the approximation of muscle activity in several regions arranged radially around user’s forearm. The intensity is represented as a polygon on a polar plane. To estimate current electrode band orientation, the user is asked to perform a certain gesture. Recorded activity is then rotated to minimise area discrepancy in each region between current occurrence and a stored pattern calculated at a known orientation. Nine gestures were considered during the preliminary assessment phase, three gestures (fist, flexion, and extension) with most promising results were chosen for further evaluation. Selected gestures were used for crossvalidation performed among three subjects using both subjectspecific and averaged models. Best results were achieved with extension, giving 13.1◦ mean orientation estimation error and standard deviation of 9.0◦. |  | X |
| **136** | Hand Gesture Recognition sensor based on accelerometer and gyroscope is a sensor for capturing the positions of operator hand while controlling underwater remotely operated vehicle equipped with an arm. The proposed system has an advantage in its convenience by means of no training or exercise needed for operator before using it. The key issue here is how beginner operator could use easily the underwater remotely operated robot arm without any specific training. The conventional one uses a joystick for controlling the underwater system and it is inconvenience for beginner user as well as less precision. The proposed system consists of two main part: (1) ground station and (2) underwater remotely operated robot arm. This paper proposes the development of hand gesture recognition sensor used by operator at the ground station for controlling robot arm at the underwater robot. The proposed sensor uses accelerometers and gyroscopes installed in elbow, forearm, and wrist. These devices measure 3D position of each joints for constructing 3D position of hand. We design sensor’s casing for its convenience of use by using CAD software. Each sensor is connected by Arduino Nano microcontroller having compact circuit and embedded it into sensor’s casing. The sensors are connected to a microcontroller acting as master connected to microcontroller slave (sensor part). These sensors value are converted to 3D position by using forward kinematic. The forward kinematic values are sent to the underwater robot by using a wire utilizing Pulse Position Signal. Then, it converted again to servo’s movement by using inverse kinematic. The result is operator able to control the underwater remotely robot arm by utilizing hand gesture directly. The last, operator could control the robot gripper based on flex sensor installed in operator’s fingers. The accuracy of the sensor has been tested under laboratory condition, it has 98% of accuracy. |  | X |
| **137** | This demonstration includes a hand gesture recognition (HGR) wristband, which can decoding the information in surface electromyography (sEMG) and micro-inertial measurement unit μ-IMU. With the complementary information from IMU, the wristband only needs two pairs of sEMG electrodes, which significantly improves the user’s experiences. A capacitive coupled body channel communication (CC-BCC) module  is also implemented in the system for wireless communication without antennas for low-power and security concerns. In this demonstration, visitors can intuitively understand the benefit and application of body channel communication for body area network. |  | X |
| **138** | Concentric tube robots have been a popular study for minimially invasive surgical procedures because of their narrow diameters and curvature. To ensure safety and reliability, the surgeon is meant to control this robot through teleoperation. Current researchers use haptic devices; however, little research has been done at investigating alternative methods that could have more simplicity, controlability, portability or humanmachine intiutiveness. Many gaming devices such as the Kinect or Leap Motion have become commercialised human-machine interfaces that are also gaining popularity in medical research for being touchless and intuitive. The work demonstrated in this paper investigates the implementation of a hand gesture visual tracking system, the hand to machine mapping algorithms and the practicality of this solution as being a teleoperation system for concentric tube robots. |  | X |

**References**

1. Haitham Badi; Alaa Hamza; Sabah Hasan, (2017), “New Method for Optimization of Static Hand Gesture Recognition”, Intelligent Systems Conference (IntelliSys), IEEE, PP 542 - 544.
2. Weiguo Zhou; Congyi Lyu; Xin Jiang; Peng Li; Haoyao Chen; Yun-Hui Liu, (2017), “Real-time Implementation of Vision-based Unmarked Static Hand Gesture Recognition with Neural Networks based on FPGAs”, International Conference on Robotics and Biomimetics (ROBIO), IEEE, PP 1026 - 1031.
3. Fuchang Yang, Hijian Shi, (2016), “Research on Static Hand Gesture Recognition Technology for Human Computer Interaction System”, International Conference on Intelligent Transportation, Big Data & Smart City, IEEE, PP 459-463.
4. Tejashree P. Salunke S .D. Bharkad, (2017), “Power Point Control Using Hand Gesture Recognition Based on Hog Feature Extraction And K-NN Classification”, International Conference on Computing Methodologies and Communication, IEEE, PP 1151-1155.
5. Himadri Nath Saha, Shinjini Ray, Sudipta Saha, Sayan Tapadar, and Suhrid Krishna Chatterjee, (2018), “A Machine Learning Based Approach for Hand Gesture Recognition using Distinctive Feature Extraction”, Computing and Communication Workshop and Conference, IEEE, PP 91-98.
6. Rosalina, Lita Yusnita, Nur Hadisukmana, R.B Wahyu, Rusdianto Roestam, Yuyu Wahyu, (2017), “Implementation of Real-Time Static Hand Gesture Recognition using Artificial Neural Network”, Computer Applications and Information Processing Technology, IEEE, PP 1-6.
7. Oinam Robita Chanu, Anushree Pillai, Spandan Sinha, Piyanka Das, (2017), “Comparative Study for Vision Based and Data Based Hand Gesture Recognition Technique”, International Conference on Intelligent Communication and Computational Techniques, IEEE, PP 26-31.
8. Jian Zhao, Jingna Mao, Guijin Wang, Huazhong Yang, and Bo Zhao, (2017), “A Miniaturized Wearable Wireless Hand Gesture Recognition System Employing Deep-Forest Classifier”, Biomedical Circuits and Systems Conference, IEEE, PP 1-4.
9. Jinxing Yang; Jianhong Pan; Jun Li, (2017), “sEMG-Based Continuous Hand Gesture Recognition Using GMM-HMM and Threshold Model”, International Conference on Robotics and Biomimetics (ROBIO), IEEE, PP 1509 - 1514.
10. Ibrahim Alnujaim; Hashim Alali; Faisal Khan; Youngwook Kim, (2018), “Hand Gesture Recognition Using Input Impedance Variation of Two Antennas with Transfer Learning”, Sensors Journal, IEEE, Volume 18, PP 4129 - 4135.
11. Yuhui Zhu; Shuo Jiang; Peter B. Shull, (2018), “Wrist-worn hand gesture recognition based on barometric pressure sensing”, International Conference on Wearable and Implantable Body Sensor Networks (BSN), IEEE, PP 181 – 184.
12. Qingrui Zhang; Mingqiang Yang; Qinghe Zheng; Xinxin Zhang, (2017), “Segmentation of Hand Gesture Based on Dark Channel Prior in Projector-Camera System”, International Conference on Communications in China (ICCC), IEEE, PP 1-6.
13. David Valencia Redrovan; Donghan Kim, (2018), “Hand Gestures Recognition using Machine Learning for control of Multiple quadrotors”, Sensors Applications Symposium (SAS), IEEE, PP 1-6.
14. Hari Prabhat Gupta; Haresh S. Chudgar; Siddhartha Mukherjee; Tanima Dutta; Kulwant Sharma, (2016), “A Continuous Hand Gestures Recognition Technique for Human-Machine Interaction Using Accelerometer and Gyroscope Sensors”, Sensors Journal, IEEE, Volume 16, PP 6425 - 6432.
15. Chenyang Zhang; Yingli Tian; Matt Huenerfauth, (2016), “Multi-modality American Sign Language recognition”, International Conference on Image Processing (ICIP), IEEE, PP 2881 - 2885.
16. Sungho Shin; Wonyong Sung, (2016), “Dynamic Hand Gesture Recognition for Wearable Devices with Low Complexity Recurrent Neural Networks”, International Symposium on Circuits and Systems (ISCAS), IEEE, PP 2274 - 2277.
17. Xiang Gao, Lei Shi, Qiang Wang, (2017), “The design of robotic wheelchair control system based on hand gesture control for the disabled”, International Conference on Robotics and Automation Sciences, IEEE, PP 30 – 34.
18. Javier Orlando Pinzón Arenas, Paula Catalina Useche Murillo, Robinson Jiménez Moreno, (2017), “Convolutional neural network architecture for hand gesture recognition”, International Conference on Electronics, Electrical Engineering and Computing, IEEE, PP 1-6.
19. C. Jose L. Flores, A. E. Gladys Cutipa, R. Lauro Enciso, (2017), “Application of convolutional neural networks for static hand gestures recognition under different invariant features”, International Conference on Electronics, Electrical Engineering and Computing, IEEE, PP 1-4.
20. Marco E. Benalcázar, Andrés G. Jaramillo, Jonathan; A. Zea, Andrés Páez, Víctor Hugo Andaluz, (2017), “Hand gesture recognition using machine learning and the Myo armband”, European Signal Processing Conference, IEEE, PP 1040-1044.
21. Aditya Tewari, Bertram Taetz, Frederic Grandidier, Didier Stricker, (2017), “A Probabilistic Combination of CNN and RNN Estimates for Hand Gesture Based Interaction in Car”, International Symposium on Mixed and Augmented Reality (ISMAR-Adjunct), IEEE, PP 1-6.
22. Rytis Augustauskas, Arunas Lipnickas, (2017), “Robust hand detection using arm segmentation from depth data and static palm gesture recognition”, International Conference on Intelligent Data Acquisition and Advanced Computing Systems: Technology and Applications, IEEE, PP 664-667.
23. Vimonhak Sombandith, Aranya Walairacht, Somsak Walairacht, (2017), “Hand gesture recognition for Lao alphabet sign language using HOG and correlation”, International Conference on Electrical Engineering/Electronics, Computer, Telecommunications and Information Technology, IEEE, PP 649-651.
24. Yuntao Ma, Yuxuan Liu, Ruiyang Jin, Xingyang Yuan, Raza Sekha, Samuel Wilson, Ravi Vaidyanathan, (2017), “Hand gesture recognition with convolutional neural networks for the multimodal UAV control”, IEEE, PP 198-203.
25. Peijun Bao, Ana I. Maqueda, Carlos R. del-Blanco, Narciso García, (2017), “Tiny Hand Gesture Recognition without Localization via a Deep Convolutional Network”, IEEE Transactions on Consumer Electronics, Volume 63, PP 251-257.
26. Yuta Sugiura, Fumihiko Nakamura, Wataru Kawai, Takashi Kikuchi, Maki Sugimoto, (2017), “Behind the palm: Hand gesture recognition through measuring skin deformation on back of hand by using optical sensors”, Conference of the Society of Instrument and Control Engineers of Japan, IEEE, PP 1082-1087.
27. Kuang-Yow Lian, Chun-Chieh Chiu, Yong-Jie Hon, Wen-Tsai Sung, (2017), “Wearable armband for real time hand gesture recognition”, International Conference on Systems, Man, and Cybernetics, IEEE, PP 2992-2995.
28. Eko Pramunanto; Surya Sumpeno; Rafiidha Selyna Legowo, (2017), “Classification of hand gesture in Indonesian sign language system using Naive Bayes”, International Seminar on Sensors, Instrumentation, Measurement and Metrology, IEEE, PP 187 – 191.
29. Jozef Goga; Slavomír Kajan, (2017), “Hand gesture recognition using 3D sensors”, International Symposium ELMAR, IEEE, PP 181 – 184.
30. S. Siji Rani; K. J. Dhrisya; M. Ahalyadas, (2017), International Conference on Advances in Computing, Communications and Informatics (ICACCI), IEEE, PP 1500 – 1505.
31. Jakub Tomczyński; Tomasz Mańkowski; Piotr Kaczmarek, (2017), “Influence of sEMG electrode matrix configuration on hand gesture recognition performance”, Signal Processing: Algorithms, Architectures, Arrangements, and Applications (SPA), IEEE, PP 42 – 47.
32. Piotr Kaczmarek; Tomasz Mańkowski; Jakub Tomczyński, (2017), “Towards sensor position-invariant hand gesture recognition using a mechanomyographic interface”, Signal Processing: Algorithms, Architectures, Arrangements, and Applications (SPA), IEEE, PP 53 - 58.
33. Jiajun Zhang; Zhiguo Shi, (2017), “Deformable deep convolutional generative adversarial network in microwave based hand gesture recognition system”, International Conference on Wireless Communications and Signal Processing (WCSP), IEEE, PP 1 – 6.
34. Nilima Mansing Patil; S. R. Patil, (2017), “Review on real-time EMG acquisition and hand gesture recognition system”, International conference of Electronics, Communication and Aerospace Technology (ICECA), IEEE, PP 694 – 696.
35. Isack Bulugu; Zhongfu Ye; Jamal Banzi, (2017), “Higher-Order Local Autocorrelation Feature Extraction Methodology for Hand Gestures Recognition”, International Conference on Multimedia and Image Processing (ICMIP), IEEE, PP 83 – 87.
36. Yu Qiao; Zhiquan Feng; Xiaoyan Zhou; Xiaohui Yang, (2017), “Principle Component Analysis Based Hand Gesture Recognition for Android Phone Using Area Features”, International Conference on Multimedia and Image Processing (ICMIP), IEEE, PP 108 – 112.
37. Shengchang Lan; Zonglong He; Haoyu Tang; Kai Yao; Wenshuang Yuan, (2017), “A Hand Gesture Recognition System Based on 24GHz Radars”, International Symposium on Antennas and Propagation (ISAP), IEEE, PP 1-2.
38. Xi Liu; Chen Li; Lihua Tian, (2017), “Hand Gesture Recognition based on Wavelet Invariant Moments”, IEEE International Symposium on Multimedia (ISM), IEEE, PP 459 – 464.
39. Shunzhan He; Chenguang Yang; Min Wang; Long Cheng; Zedong Hu, (2017), “Hand Gesture Recognition using MYO Armband”, Chinese Automation Congress (CAC), IEEE, PP 4850 – 4855.
40. Atharva Ajit Kadethankar; Apurv Dilip Joshi, (2017), “Dynamic hand gesture recognition using Kinect”, Innovations in Power and Advanced Computing Technologies (i-PACT), IEEE, PP 1-3.
41. Marco E. Benalcázar; Cristhian Motoche; Jonathan A. Zea; Andrés G. Jaramillo; Carlos E. Anchundia; Patricio Zambrano; Marco Segura; Freddy Benalcázar Palacios; María Pérez, (2017), “Real-time hand gesture recognition using the Myo armband and muscle activity detection”, Second Ecuador Technical Chapters Meeting (ETCM), IEEE, PP 1-6.
42. Andrés G. Jaramillo; Marco E. Benalcázar, (2017), “Real-time hand gesture recognition with EMG using machine learning”, Ecuador Technical Chapters Meeting (ETCM), IEEE, PP 1-5.
43. Donq-Liang Lee; Wei-Shiuan You, (2018), “Recognition of complex static hand gestures by using the wristband-based contour features”, IET Image Processing, IEEE, Volume 12, PP 80-87.
44. P. D. S. H. Gunawardane; Nimali T. Medagedara, (2017), “Comparison of hand gesture inputs of leap motion controller & data glove in to a soft finger”, International Symposium on Robotics and Intelligent Sensors (IRIS), IEEE, PP 62 – 68.
45. Chun-Jen Tsai; Yun-Wei Tsai; Song-Ling Hsu; Ya-Chiu Wu, (2017), “Synthetic Training of Deep CNN for 3D Hand Gesture Identification”, International Conference on Control, Artificial Intelligence, Robotics & Optimization (ICCAIRO), IEEE, PP 165 – 170.
46. Chenyang Li; Xin Zhang; Lianwen Jin, (2017), “LPSNet: A Novel Log Path Signature Feature Based Hand Gesture Recognition Framework”, International Conference on Computer Vision Workshops (ICCVW), IEEE, PP 631 - 639.
47. Takuya Sakamoto; Xiaomeng Gao; Ehsan Yavari; Ashikur Rahman; Olga Boric-Lubecke; Victor M. Lubecke, (2017), “Radar-based hand gesture recognition using I-Q echo plot and convolutional neural network”, Conference on Antenna Measurements & Applications (CAMA), IEEE, PP 393 – 395.
48. Rokhsana Titlee; Ashfaq Ur Rahman; Hasan U. Zaman; Hafiz Abdur Rahman, (2017), “A novel design of an intangible hand gesture controlled computer mouse using vision based image processing”, International Conference on Electrical Information and Communication Technology (EICT), IEEE, PP 1-4.
49. Rasel Ahmed Bhuiyan; Abdul Kawsar Tushar; Akm Ashiquzzaman; Jungpil Shin; Md Rashedul Islam, (2017), “Reduction of gesture feature dimension for improving the hand gesture recognition performance of numerical sign language”, International Conference of Computer and Information Technology (ICCIT), IEEE, PP 1-6.
50. Ananyaa Sharrma; Ayush Khandelwal; Kavleen Kaur; Shivani Joshi; Richa Upadhyay; Sameer Prabhu, (2017), “Vision based static hand gesture recognition techniques”, International Conference on Communication and Signal Processing (ICCSP), IEEE, PP 0705 – 0709.
51. Hafız Mohsin Abdul-Rashid; Lehmia Kiran; M. Danish Mirrani; M. Noman Maraaj, (2017), “CMSWVHG-control MS Windows via hand gesture”, International Multi-topic Conference (INMIC), IEEE, PP 1-7.
52. Erhan Akan; Hakan Tora; Baran Uslu, (2017), “Hand gesture classification using inertial based sensors via a neural network”, International Conference on Electronics, Circuits and Systems, IEEE, PP 140 – 143.
53. Shaun Canavan; Walter Keyes; Ryan Mccormick; Julie Kunnumpurath; Tanner Hoelzel; Lijun Yin, (2017), “Hand gesture recognition using a skeleton-based feature representation with a random regression forest”, International Conference on Image Processing (ICIP), IEEE, PP 2364 – 2368.
54. Xinghao Chen; Hengkai Guo; Guijin Wang; Li Zhang, (2017), “Motion feature augmented recurrent neural network for skeleton-based dynamic hand gesture recognition”, International Conference on Image Processing (ICIP), IEEE, PP 2881 – 2885.
55. Sih-Huei Chen; Ari Hernawan; Yuan-Shan Lee; Jia-Ching Wang, (2017), “Hand gesture recognition based on Bayesian sensing hidden Markov models and Bhattacharyya divergence”, International Conference on Image Processing (ICIP), IEEE, PP 3535 – 3539.
56. Yifan Zhang; Congqi Cao; Jian Cheng; Hanqing Lu, (2018), “EgoGesture: A New Dataset and Benchmark for Egocentric Hand Gesture Recognition”, Transactions on Multimedia, IEEE, Volume 20, PP 1038 – 1050.
57. S. Reshna; M. Jayaraju, (2017), “Spotting and Recognition of Hand Gesture for Indian Sign Language Recognition System with Skin Segmentation and SVM”, International Conference on Wireless Communications, Signal Processing and Networking (WiSPNET), IEEE, PP 386 – 390.
58. Rania A. Elsayed; Mohammed S. Sayed; Mahmoud I. Abdalla, (2017), “Hand Gesture Recognition Based on Dimensionality Reduction of Histogram of Oriented Gradients”, Japan-Africa Conference on Electronics, Communications and Computers (JAC-ECC), IEEE, PP 119 – 122.
59. Biyao Shao; Yifeng Xie; Hongnan Yang; Yatong Jiang; Chenggang Yan; Hongtao Xie; Yangang Wang, (2017), “A New Dataset for Hand Gesture Estimation”, Global Conference on Signal and Information Processing (GlobalSIP), IEEE, PP 1388 – 1392.
60. Said Yacine Boulahia; Eric Anquetil; Franck Multon; Richard Kulpa, (2017), “Dynamic hand gesture recognition based on 3D pattern assembled trajectories”, International Conference on Image Processing Theory, Tools and Applications (IPTA), IEEE, PP 1-6.
61. Yiwen He; Jianyu Yang; Zhanpeng Shao; Youfu Li, (2017), “Salient Feature Point Selection for Real Time RGB-D Hand Gesture Recognition”, International Conference on Real-time Computing and Robotics (RCAR), IEEE, PP 103 – 108.
62. Zengshan Tian; Jiacheng Wang; Xiaolong Yang; Mu Zhou, (2018), “WiCatch: A Wi-Fi Based Hand Gesture Recognition System”, IEEE Access, Volume 6, PP 16911 – 16923.
63. Fabian Sachara; Thomas Kopinski; Alexander Gepperth; Uwe Handmann, (2017), “Free-hand Gesture Recognition with 3D-CNNs for In-car Infotainment Control in Real-time”, International Conference on Intelligent Transportation Systems (ITSC), IEEE, PP 959 – 964.
64. Vivek D. Lad; Ramesh M. Kagalkar, (2017), “Methodology for Real Time Hand Gesture Recognition and Generating Text Description Using Histogram Techniques”, International Conference on Intelligent Computing and Control (I2C2), IEEE, PP 1-7.
65. Anna Yang; Sung Moon Chun; Jae-Gon Kim, (2018), “Detection and Recognition of Hand Gesture for Wearable Applications in IoMT”, International Conference on Advanced Communication Technology (ICACT), IEEE, PP 1-1.
66. Alvi Mahadi; Fatema Tuj Johora; Mohammad Abu Yousuf, (2016), “An Efficient Approach of Training Artificial Neural Network to Recognize Bengali Hand Sign”, International Conference on Advanced Computing (IACC), IEEE, PP 152 – 157.
67. Oyndrila De; Puskar Deb; Sagnik Mukherjee; Sayantan Nandy; Tamal Chakraborty; Sourav Saha, (2016), “Computer Vision Based Framework for Digit Recognition by Hand Gesture Analysis”, Information Technology, Electronics and Mobile Communication Conference (IEMCON), IEEE, PP 1-5.
68. Yuefeng Li; Keshi He; Xueli Sun; Honghai Liu, (2016), “Human-machine Interface Based on Multi-channel Single-element Ultrasound Transducers: A Preliminary Study”, International Conference on e-Health Networking, Applications and Services (Healthcom), IEEE, PP 1-6.
69. Vladislava Bobić; Predrag Tadić; Goran Kvaščev, (2016), “Hand gesture recognition using neural network based techniques”, Symposium on Neural Networks and Applications (NEUREL), IEEE, PP 1-4.
70. A. L. P Madushanka; R. G. D. C Senevirathne; L. M. H Wijesekara; S. M. K. D Arunatilake; K. D Sandaruwan, (2016), “Framework for Sinhala Sign Language Recognition and Translation Using a Wearable Armband”, International Conference on Advances in ICT for Emerging Regions (ICTer), IEEE, PP 49 – 57.
71. Deepali Naglot; Milind Kulkarni, (2016), “Real Time Sign Language Recognition using the Leap Motion Controller”, International Conference on Inventive Computation Technologies (ICICT), IEEE, Volume 3, PP 1-5.
72. Srinidhi Hegde; Ramakrishna Perla; Ramya Hebbalaguppe; Ehtesham Hassan, (2016), “GestAR: Real Time Gesture Interaction for AR with Egocentric View”, International Symposium on Mixed and Augmented Reality (ISMAR-Adjunct), IEEE, PP 262-267.
73. Washef Ahmed; Kunal Chanda; Soma Mitra, (2016), “Vision Based Hand Gesture Recognition Using Dynamic Time Warping for Indian Sign Language”, International Conference on Information Science (ICIS), IEEE, PP 120-125.
74. Ananta Srisuphab; Piyanuch Silapachote, (2016), “Artificial Neural Networks for Gesture Classification with Inertial Motion Sensing Armbands”, 10 Conference (TENCON), IEEE, PP 1-5.
75. Rajeshree Rokade-Shinde; Jayashree Sonawane, (2016), “Dynamic Hand Gesture Recognition”, International Conference on Signal and Information Processing (IConSIP), IEEE, PP 1-6.
76. Ashish S. Nikam; Aarti G. Ambekar, (2016), “Bilingual Sign Recognition Using Image Based Hand Gesture Technique for Hearing and Speech Impaired People”, International Conference on Computing Communication Control and automation (ICCUBEA), IEEE, PP 1-6.
77. Danling Lu; Yuanlong Yu; Huaping Liu, (2016), “Gesture Recognition Using Data Glove: An Extreme Learning Machine Method”, International Conference on Robotics and Biomimetics (ROBIO), IEEE, PP 1349 – 1354.
78. Rishabh Sharma; Raj Shikher; Nutan V. Bansode; Prachi R. Rajarapollu, (2016), “Interactive Projector Screen with Hand Detection Using Gestures”, International Conference on Automatic Control and Dynamic Optimization Techniques (ICACDOT), IEEE, PP 574 – 577.
79. Jessie R. Balbin; Dionis A. Padilla; Felicito S. Caluyo; Janette C. Fausto; Carlos C. Hortinela; Cyrel O. Manlises; Christine Kate S. Bernardino; Ezra G. Fiñones; Lanuelle T. Ventura, (2016), “Sign Language Word Translator Using Neural Networks for the Aurally Impaired as a Tool for Communication”, International Conference on Control System, Computing and Engineering (ICCSCE), IEEE, PP 425 – 429.
80. Ashish S. Nikam; Aarti G. Ambekar, (2016), “Sign Language Recognition Using Image Based Hand Gesture Recognition Techniques”, International Conference on Green Engineering and Technologies (IC-GET), IEEE, PP 1-5.
81. Lalit Kane; Pritee Khanna, (2017), “Vision-Based Mid-Air Unistroke Character Input Using Polar Signatures”, Transactions on Human-Machine Systems, IEEE, Volume 47, PP 1077 – 1088.
82. Devendrakumar H. Pal; S. M. Kakade, (2016), “Dynamic Hand Gesture Recognition Using Kinect Sensor”, International Conference on Global Trends in Signal Processing, Information Computing and Communication (ICGTSPICC), IEEE, PP 448 – 453.
83. C. V Soumya; Muzameel Ahmed, (2017), “Artificial Neural Network Based Identification and Classification of Images of Bharatanatya Gestures”, International Conference on Innovative Mechanisms for Industry Applications (ICIMIA), IEEE, PP 162 – 166.
84. Hang Zhao; Jiangping Hu; Yuping Zhang; Hong Cheng, (2017), “Hand Gesture Based Control Strategy for Mobile Robots”, Chinese Control And Decision Conference (CCDC), IEEE, PP 5868 – 5872.
85. Shome Subhra Das, (2017), “Detection of Self Intersection in Synthetic Hand Pose Generators”, International Conference on Machine Vision Applications (MVA), IEEE, PP 354 – 357.
86. Marlon Oliveira; Alistair Sutherland; Mohamed Farouk, (2016), “Two-stage PCA with Interpolated Data for Hand Shape Recognition in Sign Language”, Applied Imagery Pattern Recognition Workshop (AIPR), IEEE, PP 1-4.
87. Nabeel Siddiqui; Rosa H. M. Chan, (2017), “A Wearable Hand Gesture Recognition Device Based On Acoustic Measurements At Wrist”, International Conference of the IEEE Engineering in Medicine and Biology Society (EMBC), IEEE, PP 4443 – 4446.
88. Anshal Joshi; Heidy Sierra; Emmanuel Arzuaga, (2017), “American Sign Language Translation Using Edge Detection and Cross Correlation”, Colombian Conference on Communications and Computing (COLCOM), IEEE, PP 1-6.
89. Gi Hyun Lim; Eurico Pedrosa; Filipe Amaral; Nuno Lau; Artur Pereira; José Luís Azevedo; Bernardo Cunha, (2017), “Neural regularization jointly involving neurons and connections for robust image classification”, International Conference on Multisensor Fusion and Integration for Intelligent Systems (MFI), IEEE, PP 336 – 341.
90. T. Vedha Viyas; R. Willbert Baskar; N. Simrose Gabriel; A. Sanjive, (2017), “Hand Pantomime Apperception For Robotic Arm Control”, International conference of Electronics, Communication and Aerospace Technology (ICECA), IEEE, Volume 2, PP 120-125.
91. Zhiwen Lei; Xiaoxiao Yang; Yanzhou Gong; Weixing Huang; Jian Wang; Guigang Zhang, (2017), “A Robust Hand Cursor Interaction Method Using Kinect”, International Symposium on Multimedia (ISM), IEEE, PP 543 – 548.
92. Karthik Sivarama Krishnan; Akash Saha; Srinath Ramachandran; Shitij Kumar, (2017), “Recognition of Human Arm Gestures Using Myo Armband for the Game of Hand Cricket”, International Symposium on Robotics and Intelligent Sensors (IRIS), IEEE, PP 389-394.
93. Riya Bora; Ankita Bisht; Aradhya Saini; Tanu Gupta; Ankush Mittal, (2017), “ISL Gesture Recognition Using Multiple Feature Fusion”, International Conference on Wireless Communications, Signal Processing and Networking (WiSPNET), PP 196 – 199.
94. Stefano Sapienza; Paolo Motto Ros; David Alejandro Fernandez Guzman; Fabio Rossi; Rossana Terracciano; Elisa Cordedda; Danilo Demarchi, (2018), “On-Line Event-Driven Hand Gesture Recognition Based on Surface Electromyographic Signals”, International Symposium on Circuits and Systems (ISCAS), IEEE, PP 1-5.
95. Soukaina Chraa Mesbahi; Mohamed Adnane Mahraz; Jamal Riffi; Hamid Tairi, (2018), “Hand gesture recognition based on convexity approach and background subtraction”, International Conference on Intelligent Systems and Computer Vision (ISCV), IEEE, PP 1-5.

**Not Related to RQ: -----------------------------------------------------------------------------------------------------------------**

1. “A Data Driven In-Air-Handwriting Biometric Authentication System”
2. “A Novel Contactless Human Machine Interface based on Machine Learning”
3. “FIT-EVE&ADAM: Estimation of Velocity & Energy for Automated Diet Activity Monitoring”
4. “Sensors for Virtual Musical Environment: A Short Survey”
5. “Motion Initiated Music Ensemble with Sensors for Motor Rehabilitation”
6. “Toward Hand-Dominated Activity Recognition Systems With Wristband-Interaction Behavior Analysis”
7. “Virtual Reality Markup Framework for Generating Interactive Indoor Environment”
8. “Gesture Based Control of IRB1520ID using Microsoft’s Kinect”
9. “Comparing Techniques of Segmenting Hand Region”
10. “Analysis and Implementation of Simple Gesture Recognition Algorithms using MATLAB”
11. “The Medical Service Robot Interaction Based on Kinect”
12. “Wireless Head Gesture Controlled Wheel Chair for Disable Persons”
13. “Design of multi-degree-of-freedom spring mechanisms: biomimetic approach”
14. “Multi-Order Statistical Descriptors for Real-Time Face Recognition and Object Classification”
15. “Sensor Fusion of Leap Motion Controller and Flex Sensors Using Kalman Filter for Human Finger Tracking”
16. “Intuitive Human-Device Interaction for Video Control and Feedback”
17. “Pan-and-Tilt Self-Portrait System Using Gesture Interface”
18. “SeleCon: Scalable IoT Device Selection and Control Using Hand Gestures”
19. “MOTION TRACKING FOR GESTURE ANALYSIS IN SPORTS”
20. “Linear and Rotational Air Gesture Detection using Optical Sensors Setup in Automotive Infotainment System”
21. “Smart Surface: RFID-Based Gesture Recognition Using k-Means Algorithm”
22. “Wireless Hand Gesture Recognition Based on Continuous-Wave Doppler Radar Sensors”
23. “Detection of Chewing from Piezoelectric Film Sensor Signals using Ensemble Classifiers”
24. “Development of a Robotic Arm and implementation of a control strategy for gesture recognition through Leap Motion device.”
25. “Towards Teleoperation-based Interactive Learning of Robot Kinematics using a Mobile Augmented Reality Interface on a Tablet”
26. “Deep Dynamic Neural Networks for Multimodal Gesture Segmentation and Recognition”
27. “Nonparametric Feature Matching Based Conditional Random Fields for Gesture Recognition from Multi-Modal Video”
28. “Transradial Amputee Gesture Classification Using an Optimal Number of sEMG Sensors: An Approach Using ICA Clustering”
29. “A Multisensor Technique for Gesture Recognition Through Intelligent Skeletal Pose Analysis”
30. “ModDrop: Adaptive Multi-Modal Gesture Recognition”
31. Harpreet Kaur; Jyoti Rani, (2016), “A review: Study of various techniques of Hand gesture recognition”, International Conference on Power Electronics, Intelligent Control and Energy Systems (ICPEICES), IEEE, PP 1-5.
32. Sungpill Choi; Jinsu Lee; Kyuho Lee; Hoi-Jun Yoo, (2018), “A 9.02mW CNN-Stereo-Based Real-Time 3D Hand-Gesture Recognition Processor for Smart Mobile Devices”, International Solid - State Circuits Conference - (ISSCC), IEEE, PP 220 – 222.
33. Ujwal Bachiraju Venkata Satya; Venkat R Peddigari, (2017), “Continuous, Robust Hand Gesture Recognition for Embedded Devices”, International Conference on Consumer Electronics-Asia (ICCE-Asia), IEEE, PP 117 – 120.
34. R. Ramachandiran; P. Victer Paul; K. Ramapraba; M. Vignesh; R. Hariprasath, (2017), “Control the CPU power with hand gesture recognition using IR-pair sensor”, International Conference on Computation of Power, Energy Information and Communication (ICCPEIC), IEEE, PP 253 – 256.
35. Gergely Sziladi; Tibor Ujbanyi; Jozsef Katona; Attila Kovari, (2017), “The analysis of hand gesture based cursor position control during solve an IT related task”, International Conference on Cognitive Infocommunications, IEEE, PP 000413 – 000418.
36. Hasan U. Zaman; Asif Alam Joy; Khan Mohammed Akash; Safwan Talukder Fayad, (2017), “A simple and effective way of controlling a robot by hand gesture”, International Conference on Intelligent Computing and Control Systems, IEEE, PP 330 – 333.
37. Masayuki Miyasugi; Hideo Akaike; Yasuichi Nakayama; Hiroyasu Kakuda, (2017), “Implementation and Evaluation of Multi-User Mind Map Authoring System using Virtual Reality and Hand Gestures”, Global Conference on Consumer Electronics (GCCE), IEEE, PP 1-5.
38. Nilima Mansing Patil; S. R. Patil, (2017), “Review on Real-Time EMG Acquisition and Hand Gesture Recognition system”, International conference of Electronics, Communication and Aerospace Technology (ICECA), IEEE, PP 694 – 696.
39. Hee-Kwon Kim; Jisu Lee; ChoRong Yu; HeeSook Shin; Youn-Hee Gil; Hyung-Keun Jee, (2017), “3D hand gestures calibration method for multi-display by using a depth camera”, International Conference on Information and Communication Technology Convergence (ICTC), IEEE, PP 1044 – 1046.
40. Jakub Tomczyński; Tomasz Mańkowski; Piotr Kaczmarek, (2017), “Localisation method for sEMG electrode array, towards hand gesture recognition HMI development”, Signal Processing: Algorithms, Architectures, Arrangements, and Applications (SPA), IEEE, PP 48 – 52.
41. Ronny Mardiyanto; Mochamad Fajar Rinaldi Utomo; Djoko Purwanto; Heri Suryoatmojo, (2017), “Development of hand gesture recognition sensor based on accelerometer and gyroscope for controlling arm of underwater remotely operated robot”, International Seminar on Intelligent Technology and Its Applications, IEEE, PP 329-333.
42. Jingna Mao; Jian Zhao; Guijin Wang; Huazhong Yang; Bo Zhao, (2017), “Live Demonstration: A Hand Gesture Recognition Wristband employing Low Power Body Channel Communication”, Biomedical Circuits and Systems Conference (BioCAS), IEEE, PP 1-1.
43. Andrew Razjigaev; Ross Crawford; Jonathan Roberts; Liao Wu, (2017), “Teleoperation of a Concentric Tube Robot through Hand Gesture Visual Tracking”, International Conference on Robotics and Biomimetics (ROBIO), IEEE, PP 1175 - 1180.
