# Supplementary material for: A systematic review on hand gesture recognition techniques, challenges and applications
Source: PeerJ Comput Sci. 2019 Sep 16;5:e218. doi: 10.7717/peerj-cs.218 (PMC7924500; doi:10.7717/peerj-cs.218)
Supplement: Supplemental Information 2 [file peerj-cs-05-218-s002.pdf]

PRISMA 2009 Checklist

Section/topic	#	Checklist item	Reported on page #
**TITLE**			
Title	1	Identify the report as a systematic review, meta-analysis, or both.	Page 1: "A Systematic Review on Hand Gesture Recognition Techniques, Challenges and Applications."
**ABSTRACT**			
Structured summary	2	Provide a structured summary including, as applicable: background; objectives; data sources; study eligibility criteria, participants, and interventions; study appraisal and synthesis methods; results; limitations; conclusions and implications of key findings; systematic review registration number.	Page 1: "Background. With the development of today's technology, and as humans tend to naturally use hand gestures in their communication process to clarify their intentions, hand gesture recognition is considered to be an important part of Human Computer Interaction (HCI), which gives the computers the ability of capturing and interpreting hand gestures, and executing commands afterwards. The aim of this study is to perform a systematic literature review for identifying the most prominent techniques, applications and challenges in hand gesture recognition. Methodology. To conduct this systematic review, we have screened 560 papers retrieved from IEEE Explore published from the year 2016 to 2018, in the searching process keywords such as "hand gesture recognition" and "hand gesture techniques" have been used. However, to focus the scope of the study 465 papers have been excluded. Only the most relevant hand gesture recognition works to the research questions, and the well-organized papers have been studied. Results. The results of this paper can be summarized as the following; the surface electromyography (sEMG) sensors with wearable hand gesture devices were the most acquisition tool used in the work studied, also Artificial Neural Network (ANN) was the most applied classifier, the most popular application was using hand gestures for sign language, the dominant environmental surrounding factor that affected the accuracy was the background color, and finally the problem of overfitting in the datasets was highly experienced. Conclusions. The paper will discuss the gesture acquisition methods, the feature extraction process, the classification of hand gestures, the applications that were recently proposed, the challenges that face researchers in the hand gesture recognition process, and the future of hand gesture recognition. We shall also introduce the most recent research from the year 2016 to the year 2018 in the field of hand gesture recognition for the first time."
**INTRODUCTION**			
Rationale	3	Describe the rationale for the review in the context of what is already known.	Page 2: "With the development of today's technology, and as humans tend to naturally use hand gestures in their communication process to clarify their intentions, hand gestures can play an important role in interchanging information between humans and computers (Aashni et al., 2017)."<br>Page 3: "In this research, we survey the latest researches that were done on hand gesture recognition. We shall also compare the different techniques, applications, and challenges presented by the surveyed work. The reason why the most recent research articles from IEEE database were chosen to be studied is that we wanted to construct a valid base of the current situation and technologies of hand gesture recognition.

			Furthermore, the articles published by IEEE in the year of 2016 to 2018 were considered to increase the intensity and focus of this study, and because the recent works were not sufficiently studied before, where the older ones were studied and compared more such as in (Rafiqul & Noor, 2012; Arpita. G. & S., 2013; Deepali & J., 2012). The contribution of this research can be summarized as the following: 1. Introducing the most recent researches from the year 2016 to the year 2018 in the field of hand gesture recognition for the first time.2. Comparing the different techniques proposed, applications applied, and challenges discussed in the current available technology of hand gestures recognition."
Objectives	4	Provide an explicit statement of questions being addressed with reference to participants, interventions, comparisons, outcomes, and study design (PICOS).	Page 3: "This review is guided by the following 3 research questions: 1. What are the techniques used in hand gesture recognition? 2. What are the domains and applications that make use of hand gesture recognition? 3. What are the challenges in hand gesture recognition applications?"
**METHODS**			
Protocol and registration	5	Indicate if a review protocol exists, if and where it can be accessed (e.g., Web address), and, if available, provide registration information including registration number.	-
Eligibility criteria	6	Specify study characteristics (e.g., PICOS, length of follow-up) and report characteristics (e.g., years considered, language, publication status) used as criteria for eligibility, giving rationale.	Page 4: "Two main keywords were used for the search process: hand gesture recognition and hand gesture techniques, other keywords were also used (such as hand gestures, hand gesture systems, hand gesture classification, hand gesture feature extraction, and sign language recognition) to include synonyms, where the keywords focused on the domain of research questions."
Information sources	7	Describe all information sources (e.g., databases with dates of coverage, contact with study authors to identify additional studies) in the search and date last searched.	Page 4: "The first step of this systematic literature review is gathering all retrieved documents from the year 2016 to the year 2018, where the process of screening includes downloading the papers published on IEEE Explore, and reading their titles and abstracts."
Search	8	Present full electronic search strategy for at least one database, including any limits used, such that it could be repeated.	Page 4: "Two main keywords were used for the search process: hand gesture recognition and hand gesture techniques, other keywords were also used (such as hand gestures, hand gesture systems, hand gesture classification, hand gesture feature extraction, and sign language recognition) to include synonyms, where the keywords focused on the domain of research questions."
Study selection	9	State the process for selecting studies (i.e., screening, eligibility, included in systematic review, and, if applicable, included in the meta-analysis).	Page 4: "All 244 papers were reviewed and evaluated in reference to the research questions, where authors read the abstracts and evaluated whether they can find an answer to any of the core questions of this review, for example some of the papers excluded were discussing the electrical aspect of hand gesture acquisition methods, this process excluded 100 papers out of 244. Full papers of 144 potentially relevant references were selected for further examination. After reading the full papers, 49 poorly organized documents that do not have a clear methodology justification were removed due to lack of validation and weakness of results justification, for example some of the works excluded did not explain why they chose their approach of acquisition or

			classification in comparison with all the possible approaches available in the recent technology, or did not explain why their proposed approach performed in the way they provide. Only 95 papers were selected to be included in this paper. Overall, the selection was made by the authors based on two criteria; relevance to the research questions, organization and form of writing of the papers studied. Classification of the selected paper is demonstrated in Table 1 which also shows the number of papers (intersected) included in each class and subclass. 180 papers were relevant to Hand Gesture Techniques, 53 papers were relevant to Hand Gesture Recognition Applications, and 31 papers were relevant to Hand Gesture Recognition Challenges."
Data collection process	10	Describe method of data extraction from reports (e.g., piloted forms, independently, in duplicate) and any processes for obtaining and confirming data from investigators.	Page 4: "Literature included 1,045 studies. After removing duplicates 560 titles were screened. The duplication occurs because the same work was retrieved more than once when multiple keywords were used. Then 316 papers were excluded for having titles with no relevance to the review questions in general, where titles were scanned and processed by authors to specify whether the works are pertinent to any chosen subcategory of hand gesture recognition. The next step was screening abstracts of all 244 retrieved documents."
Data items	11	List and define all variables for which data were sought (e.g., PICOS, funding sources) and any assumptions and simplifications made.	Page 4: "The first step of this systematic literature review is gathering all retrieved documents from the year 2016 to the year 2018, where the process of screening includes downloading the papers published on IEEE Explore, and reading their titles and abstracts. Two main keywords were used for the search process: hand gesture recognition and hand gesture techniques, other keywords were also used (such as hand gestures, hand gesture systems, hand gesture classification, hand gesture feature extraction, and sign language recognition) to include synonyms, where the keywords focused on the domain of research questions."
Risk of bias in individual studies	12	Describe methods used for assessing risk of bias of individual studies (including specification of whether this was done at the study or outcome level), and how this information is to be used in any data synthesis.	-
Summary measures	13	State the principal summary measures (e.g., risk ratio, difference in means).	-
Synthesis of results	14	Describe the methods of handling data and combining results of studies, if done, including measures of consistency (e.g., I^2) for each meta-analysis.	Page 5: "The procedure of hand gesture recognition is conducted by executing several steps as illustrated in Fig. 3; image frame acquisition or gesture acquisition is to capture the human hand gesture image by the computer (Ananya, 2015). This could be done using vision-based recognition where no special gadgets are required, and a web camera or a depth camera is used, furthermore special tools can be utilized such as wired or wireless gloves that detect the movements of the user hand, and motion sensing input devices (Kinect from Microsoft, Leap Motion, …etc.) that captures the hand gestures and motions. Hand tracking process is the ability of the computer to trace the user hand and separate it from the background and from the surrounding objects (Ananya, 2015). This can be done using multi-scale color feature hierarchies that gives the users hand and the background different shades of colors to be able to identify and

			remove the background, or by using clustering algorithms that are capable of treating each finger as a cluster and removing the empty spaces in-between them. The features extracted change from one application to another, some of the features that could be taken into consideration are: fingers status, thumb status, skin color, alignments of fingers, and the palm position (Ananya, 2015). These features and other features can be extracted using several methods available, such as Fourier descriptor method which captures the palm, the fingers and the finger tips, or centroid method which captures the essential structure of the hand. The features extracted are then sent to training and testing the classification algorithm (such as Artificial Neural Networks (ANN), K-nearest neighbor (KNN), Naive Bayes (NB), Support Vector Machine (SVM), …etc.) to reach the output gesture, for example the output gesture in a simple case can contain two classes to detect only two gestures such as open and closed hand gestures. In this section we will be discussing the papers that were extracted in reference to the research questions in details, where Fig. 4 demonstrates the results of this review, answering each research question by showing the most popular method used in each subcategory.”

Section/topic	#	Checklist item	Reported on page #
Risk of bias across studies	15	Specify any assessment of risk of bias that may affect the cumulative evidence (e.g., publication bias, selective reporting within studies).	-
Additional analyses	16	Describe methods of additional analyses (e.g., sensitivity or subgroup analyses, meta-regression), if done, indicating which were pre-specified.	-
**RESULTS**			
Study selection	17	Give numbers of studies screened, assessed for eligibility, and included in the review, with reasons for exclusions at each stage, ideally with a flow diagram.	Page 4: “A summary of the identification and selection of articles for inclusion in this review is presented in Fig. 1, according to the PRISMA statement (Moher et al., 2009).”
Study characteristics	18	For each study, present characteristics for which data were extracted (e.g., study size, PICOS, follow-up period) and provide the citations.	Page 5: “The focus of our review in this section is on different gesture acquisition methods that has been used, the features considered for different applications and the feature extraction methods applied on the images to abstract those features, and finally the classification algorithms that has been recently used for classifying hand gestures.”<br>Page 5: “Image hand gesture acquisition, as illustrated in Fig. 5 from (Kinect, 2019) is to capture the human hand gesture image by the computer (Ananya, 2015). This could be done using vision-based recognition where no special gadgets are required and a web camera or a depth camera is used, furthermore special tools can be utilized such as wired or wireless gloves that detect the movements of the user hand, and motion sensing input devices (Kinect from Microsoft, Leap Motion, …etc.) that capture the hand gestures and motions as showed in Fig. 5.”

Risk of bias within studies	19	Present data on risk of bias of each study and, if available, any outcome level assessment (see item 12).	-
Results of individual studies	20	For all outcomes considered (benefits or harms), present, for each study: (a) simple summary data for each intervention group (b) effect estimates and confidence intervals, ideally with a forest plot.	Page 8: "The work of (Haitham, Alaa & Sabah, 2017) applied two methods of feature extraction, a contour hand and complex Alhzat. In (Weiguo et al., 2017) feature extraction module was applied using Discrete Fourier Transform (DFT) operations of histograms (vertical and horizontal). The proposed approach of (Tejashree, Salunke & Bharkad, 2017) took the input data from a portable webcam then processed the images and after that extracted a histogram of gradients features. The objective of (Himadri et al., 2018) was to do segmentation of the hands using polygon approximation and approximate convex decomposition, then recording the unique features between various convex segments of the hand as a method of feature extraction. Regional Contrast (RC) based salient object extraction algorithm, and a method using the color statistics of image were used in (Qingrui et al., 2017). To detect the start and end points of gestures a gesture spotting algorithm was applied in (Hari et al., 2016). Experiments of (Chenyang, Yingli & Matt, 2016) followed 5 different feature extraction strategies; depth image sequence, body joints & facial landmarks, hand shapes & facial expressions/attributes. In (C. Jose et al., 2017), digital image processing techniques were used to eliminate noise, to improve the contrast under different illumination, to separate the hand from the background and to cut the region containing the hand. Histogram of Oriented Gradients (HOG) was used in (Vimonhak, Aranya & Somsak, 2017) for image processing to extract characteristics of the hand. In the work of (Nilima & S., 2017), the Accurate End Point Identification method was implemented and applied on gesture images which were captured in varying backgrounds to detect edge points and branch points and it was also applied on blurred images containing multiple objects. The authors of (Isack, Zhongfu & Jamal, 2017) employed higher order local autocorrelation (HLAC) feature extraction method."
Synthesis of results	21	Present results of each meta-analysis done, including confidence intervals and measures of consistency.	-
Risk of bias across studies	22	Present results of any assessment of risk of bias across studies (see Item 15).	-
Additional analysis	23	Give results of additional analyses, if done (e.g., sensitivity or subgroup analyses, meta-regression [see Item 16]).	-
**DISCUSSION**			
Summary of evidence	24	Summarize the main findings including the strength of evidence for each main outcome; consider their relevance to key groups (e.g., healthcare providers, users, and policy makers).	Page 20: "This paper had successfully presented the most prominent techniques, applications, and challenges in hand gesture recognition. These include the gesture acquisition methods, the feature extraction process, the classification of hand gestures, the applications that were recently proposed in the field of sign language, robotics and other fields, the environmental surroundings challenges and the datasets challenges that face researchers in the hand gesture recognition process, and the future of hand gesture recognition. The results of this paper can be summarized as the following; the surface

			electromyography (sEMG) sensors with wearable hand gesture devices were the most acquisition tools used in the works studied, also the Artificial Neural Network (ANN) was the most applied classifier, the most popular application was using hand gestures for sign language, the dominant environmental surrounding factor that affected the accuracy was the background color, and the common problem found in many of the studies was overfitting in the datasets."
Limitations	25	Discuss limitations at study and outcome level (e.g., risk of bias), and at review-level (e.g., incomplete retrieval of identified research, reporting bias).	Page 16: "The process of hand gestures recognition consists of a set of complex steps that has many possible difficulties that could stand in the way of having accurate recognition. In this section we will discuss the major environmental surroundings challenges and the training and testing dataset challenges that could occur."
Conclusions	26	Provide a general interpretation of the results in the context of other evidence, and implications for future research.	Page 19: "Despite the high performance of some of the recent methods discussed in this research, the hand gesture recognition is still an evolving topic that needs more experiments. Hand gesture recognition method also needs to be extended to cover all of the areas of information technology and artificial intelligence, such as tablets, smartphones, gaming consoles, smart televisions, laptops and desktops (Hexa, 2017). It is no doubt that hand gesture recognition is capable of enabling natural communication and intelligence into applications that humans use every day. Hand gesture recognition is employing the principle of perceptual computing and changing the methods of human computer interaction (HCI) making them less complex and more enjoyable. Applications such as home control systems, healthcare systems, gaming technologies, automobiles, televisions, home automations, and robotics are expected to be able to use hand gesture recognition to represent the communication between the user and the devices (Hexa, 2017). Furthermore, some of the applications are very sensitive and in need of having a high recognition accuracy almost close to 100%, to be able to use them without causing any damage or danger to human lives; such as applications of the health field, the transportation field, and the flight operation field. Hand gesture recognition was recently applied in some gaming consoles has increased the sales rate of these consoles (such as Xbox and PlayStation), it is expected to keep growing more and more over time. Smartphones are expected to experience the highest growth of hand gesture recognition (Hexa, 2017). Smart televisions are also expected to experience a growth in this topic and increase the purchasing rate of the latest technology using hand gestures (Hexa, 2017). The topic is expected to grow over 28% from the year 2017 to 2024 (Hexa, 2017)."
**FUNDING**			
Funding	27	Describe sources of funding for the systematic review and other support (e.g., supply of data); role of funders for the systematic review.	-

From: Moher D, Liberati A, Tetzlaff J, Altman DG, The PRISMA Group (2009). Preferred Reporting Items for Systematic Reviews and Meta-Analyses: The PRISMA Statement. PLoS Med 6(7): e1000097. doi:10.1371/journal.pmed1000097

For more information, visit: **www.prisma-statement.org**.
