# Supplementary material for: A systematic review on hand gesture recognition techniques, challenges and applications
Source: PeerJ Comput Sci. 2019 Sep 16;5:e218. doi: 10.7717/peerj-cs.218 (PMC7924500; doi:10.7717/peerj-cs.218)
Supplement: Supplemental Information 3 [file peerj-cs-05-218-s003.pdf]

The contribution of this research can be summarized as the following:

1. Introducing the most recent researches from the year 2016 to the year 2018 in the field of hand gesture recognition for the first time.
2. Comparing the different techniques proposed, applications applied, and challenges discussed in the current available technology of hand gestures recognition.
