# Supplementary material for: A systematic review on hand gesture recognition techniques, challenges and applications
Source: PeerJ Comput Sci. 2019 Sep 16;5:e218. doi: 10.7717/peerj-cs.218 (PMC7924500; doi:10.7717/peerj-cs.218)
Supplement: Supplemental Information 4 [file peerj-cs-05-218-s004.pdf]

Rationale of systematic review

To explain why this is a systematic literature review; this is one of the popular literature review types which has been applied on many fields including Human Computer Interaction. The process of the systematic literature review is illustrated in Fig. 1 "PRISMA flow diagram of the study selection process".

To explain why the area of study was from IEEE and was focused on 2016-2018; the reason why the most recent research from IEEE were chosen to be studied is that we wanted to construct a valid base of the current situation and technologies of hand gesture recognition. Furthermore, the works from IEEE in the year 2016 to 2018 were considered to increase the intensity and focus of this study, and because the recent works were not sufficiently studied before, where the older ones were studied and compared more such as in (Rafiqul & Noor, 2012; Arpita. G. & S., 2013; Deepali & J., 2012).

This review is guided by the following 3 research questions:

1. What are the techniques used in hand gesture recognition?
2. What are the domains and applications that make use of hand gesture recognition?
3. What are the challenges in hand gesture recognition applications?

To clarify the applications section; gestures are surely being explored in many applications currently due to the technological advances, however this section reflects the most commonly and recently applied gestures rather than all of the available gestures in todays' technology.

The first step of this systematic literature review is gathering all retrieved documents from the year 2016 to the year 2018, where the process of screening includes downloading the papers published on IEEE Explore, and reading their titles and abstracts.

To explain the search keywords used; Two main keywords were used for the search process: hand gesture recognition and hand gesture techniques, other keywords were also used (such as hand gestures, hand gesture systems, hand gesture classification, hand gesture feature extraction, and sign language recognition) to include synonyms, where the keywords focused on the domain of research questions. The search process was performed by both authors (Mais Yasen and Shaidah Jusoh). A summary of the identification and selection of articles for inclusion in this review is presented in Fig. 1, according to the PRISMA statement (Moher et al., 2009). Literature included 1,045 studies.

To explain what duplicate removal is; after removing duplicates 560 titles were screened. The duplication occurs because the same work was retrieved more than once when multiple keywords were used.

To explain what title scanning is; then 316 papers were excluded for having titles with no relevance to the review questions in general, where titles were scanned and processed by all authors to specify whether the works are pertinent to any chosen subcategory of hand gesture recognition.

To explain what abstract scanning is; the next step was screening abstracts of all 244 retrieved documents. All 244 papers were reviewed and evaluated in reference to the research questions, where authors read the abstracts and evaluated whether they can find an answer to any of the

core questions of this review, for example some of the papers excluded were discussing the electrical aspect of hand gesture acquisition methods, this process excluded 100 papers out of 244.

To explain what full document scanning is; full papers of 144 potentially relevant references were selected for further examination. After reading the full papers, 49 poorly organized documents that do not have a clear methodology justification were removed due to lack of validation and weakness of results justification, for example some of the works excluded did not explain why they chose their approach of acquisition or classification in comparison with all the possible approaches available in the recent technology, or did not explain why their proposed approach performed in the way they provide. Only 95 papers were selected to be included in this paper.

To address researchers involved in the process of final selection; overall, the selection was made by all authors based on two criteria; relevance to the research questions, organization and form of writing of the papers studied. Classification of the selected paper is demonstrated in Table 1 which also shows the number of papers (intersected) included in each class and subclass. 180 papers were relevant to Hand Gesture Techniques, 53 papers were relevant to Hand Gesture Recognition Applications, and 31 papers were relevant to Hand Gesture Recognition Challenges.
